# Supplementary material for: O-mannosylation of misfolded ER proteins promotes ERAD
Source: EMBO J. 2025 Dec 5;45(2):564–91. doi: 10.1038/s44318-025-00647-2 (PMC12811338; doi:10.1038/s44318-025-00647-2)
Supplement: Supplementary file 1 — Appendix [file 44318_2025_647_MOESM1_ESM.pdf]

Appendix for

O-mannosylation of misfolded proteins promotes ERAD

Leticia Lemus[1,*], Hadar Meyer[2], Ana I. Rodríguez-Rosado[1] , Maya Schuldiner[2] and Veit Goder[1,*]

[1] Dept. of Genetics, University of Seville, Av. Reina Mercedes 6, 41012 Seville, Spain.

[2] Dept. of Molecular Genetics, Meyer Bldg. Room 122, Weizmann Institute of Sciences, 76100 Rehovot, Israel

*Corresponding authors Emails:

llemus@us.es

vgoder@us.es

Table of Content:

Appendix Table S1 page 2

Appendix Table S2 page 5

Appendix Table S1. List of yeast strains used in this study.

Strain	Genotype	Reference
VGY100	BY background; MATa his3Δ1 leu2Δ0 met15Δ0 ura3Δ0	PMID: 9483801
VGY381	W303 background; MATa; leu2-3,112 trp1-1 can1-100 ura3-1 ade2-1 his3-11,15	PMID: 22977733
VGY3587	BY background; MTAx; his3Δ1 leu2Δ0 met15Δ0 ura3Δ0, can1Δ::STE2pr-spHIS5, lyp1Δ::STE3pr-LEU2	PMID: 26928762
CRY209	BY background; MATa; HA-CPY*SRR_TMD (VGc418, URA3, CEN)	this study
CRY219	BY background; MATa; GAL1P-3HA-PBN1::NAT; HA-GAS1*(VGp167, URA3, CEN)	this study
CRY222	BY background; MATa; GAL1P-3HA-PBN1::NAT; HA-GAS1*TMD (VGp176, URA3, CEN)	this study
CRY253	BY background; MATa; Δemp24::KanMX; GAL1P-3HA-PBN1::NAT; HA-CPY*SRR_TMD (VGc418, URA3, CEN)	this study
CRY255	BY background; MATa; Δdoa10::KanMX; GAL1P-3HA-PBN1::NAT; HA-CPY*SRR_TMD (VGc418, URA3, CEN)	this study
CRY267	BY background; MATa; HA-CPY* (VGc421, URA3, CEN)	this study
CRY268	BY background; MATa; GAL1P-3HA-PBN1::NAT; HA-CPY* (VGc421, URA3, CEN)	this study
CRY276	BY background; MATa; Δhrd1::HYG; GAL1P-3HA-PBN1::NAT; HA-CPY*SRR_TMD (VGc418, URA3, CEN)	this study
CRY277	BY background; MATa; Δasi1::KanMX; GAL1P-3HA-PBN1::NAT; HA-CPY*SRR_TMD (VGc418, URA3, CEN)	this study
CRY281	BY background; MATa; GAL1P-3HA-PBN1::NAT; HA-CPY*SRR_TMD (VGc418, URA3, CEN)	this study
MBY017	BY background; MATx; GFP-PBN1 (SWAT); SEC63-mCherry (VGp241, URA3, CEN)	this study
VGY301	BY background; MATa; Δire1::KanMX	this study
VGY611	W303 background; MATa; HA-GAS1*::URA3 (VGp165, integrative)	this study
VGY625	W303 background; MATa; Δemp24::HYG; HA-GAS1*::URA3 (VGp165, integrative)	this study
VGY699	W303 background; MATx; Δemp24::HYG; Δpmt1::HIS; Δpmt2::KanMX; HA-GAS1*::URA3 (VGp165, integrative)	this study
VGY1179	BY background; MATx; Δhrd1::HYG; ΔNg-CPY*FLAG (VGp381, URA3, CEN)	this study
VGY1600	W303 background; MATa; Δted1::HYG; HA-GAS1*::URA3 (VGp165, integrative)	this study
VGY1656	W303 background; MATa; Δcwh43::HYG; HA-GAS1*::URA3 (VGp165, integrative)	this study
VGY2189	BY background; MATa; HA-GAS1* (VGp167, URA3, CEN)	this study
VGY2337	BY background; MATa; GAL4P-LEU2-TMD-GAS1*(VGp161, URA3, CEN)	this study
VGY2339	BY background; MATa; Δubc7::KanMX; GAL4P-LEU2-TMD-GAS1*(VGp161, URA3, CEN)	this study
VGY2407	BY background; MATa; HA-GAS1*TMD (VGp176, URA3, CEN)	this study
VGY3152	BY background; MATa; GFP-GAS1*::URA3	this study
VGY3171	BY background; MATa; Δemp24::KanMX; GFP-GAS1*::URA3	this study
VGY3260	BY background; MATa; HA-CPY*SRR_TMD (VGc418, URA3, CEN)	this study
VGY3596	BY background; MTAx; his3Δ1 leu2Δ0 met15Δ0 ura3Δ0, can1Δ::STE2pr-spHIS5, lyp1Δ::STE3pr-LEU2; GFP-GAS1*::URA3 (VGc226, integrative)	this study
VGY3746	BY background; MTAx; Δted1::NAT; his3Δ1 leu2Δ0 met15Δ0 ura3Δ0, can1Δ::STE2pr-spHIS5, lyp1Δ::STE3pr-LEU2; GFP-GAS1*::URA3 (VGc226, integrative)	this study
VGY3865	BY background; MATa; HA-CPY* (VGc421, URA3, CEN)	this study
VGY3866	BY background; MATa; PBN1-DAmP::KanMX; HA-GAS1*TMD (VGp176, URA3, CEN)	this study
VGY3867	BY background; MATa; PBN1-DAmP::KanMX; HA-CPY*SRR_TMD (VGc418, URA3, CEN)	this study
VGY3868	BY background; MATa; PBN1-DAmP::KanMX; HA-CPY* (VGc421, URA3, CEN)	this study
VGY3874	BY background; MATx; Δpmt1::HIS; Δpmt2::KanMX; HA-CPY*SRR_TMD (VGc418, URA3, CEN)	this study

VGY3909	BY background; MATx; his3Δ1 leu2Δ0 met15Δ0 ura3Δ0, can1Δ::STE2pr-spHIS5, lyp1Δ::STE3pr-LEU2; TEF2P-KAR2ss-mCherry-PBN1::NAT; TDH3P-KAR2ss-GFP-HDEL (VGp341, URA3, CEN)	this study
VGY3963	BY background; MATx; NOP1P-KAR2ss-GFP-PBN1::URA; SEC63-mCherry (VGp242, LEU2, CEN)	this study
VGY4164	BY background; MATa; GFP-CPY*SRR_TMD (VGp468, URA3, CEN)	this study
VGY4165	BY background; MATa; PBN1-DAmP::KanMX; GFP-CPY*SRR_TMD (VGp468, URA3, CEN)	this study
VGY4207	BY background; MATa; Δpbn1::KAnMX; GFP-PBN1 (VGc476, LEU2, CEN)	this study
VGY4224	BY background; MATa; PBN1-DAmP::KanMX; HA-GAS1* (VGp167, URA3, CEN)	this study
VGY4237	BY background; MATa; HA-GAS1wt (VGp310, URA3, CEN)	this study
VGY4238	BY background; MATa; GAL1P-3HA-PBN1::NAT; HA-GAS1wt (VGp310, URA3, CEN)	this study
VGY4250	BY background; MATx; PBN1-AID*-GFP::HYG; osTIR::HIS	this study
VGY4283	BY background; MATa; PBN1-DAmP::KanMX; HA-GAS1wt (VGp310, URA3, CEN)	this study
VGY4285	BY background; MATx; Δpmt1::HIS; Δpmt2::KanMX; GFP-CPY*SRR_TMD (VGp468, URA3, CEN)	this study
VGY4289	BY background; MATa; Δpbn1::KAnMX; PBN1-MYC (VGc445, URA3, 2micron); GFP-PBN1Δ21-208 (VGc489, LEU2, CEN)	this study
VGY4290	BY background; MATa; Δpbn1::KAnMX; PBN1-MYC (VGc445, URA3, 2micron); GFP-PBN1Δ21-208 (VGc489, LEU2, CEN)	this study
VGY4295	BY background; MATa; Δpbn1::KAnMX; PBN1-MYC (VGc445, URA3, 2micron); pRS315 (LEU2, CEN)	this study
VGY4296	BY background; MATa; Δpbn1::KAnMX; PBN1-MYC (VGc445, URA3, 2micron); GFP-PBN1 (VGc476, LEU2, CEN)	this study
VGY4297	BY background; MATa; Δpbn1::KAnMX; PBN1-MYC (VGc445, URA3, 2micron); GFP-PBN1 (VGc476, LEU2, CEN)	this study
VGY4300	BY background; MATx; Δpmt1::HIS; Δpmt2::KanMX; HA-GAS1* (VGp167, URA3, CEN)	this study
VGY4308	BY background; MATa; Δpbn1::KAnMX; PBN1-MYC (VGc445, URA3, 2micron); GFP-PBN1ΔTMD (VGc490, LEU2, CEN)	this study
VGY4309	BY background; MATa; Δpbn1::KAnMX; PBN1-MYC (VGc445, URA3, 2micron); GFP-PBN1ΔTMD (VGc491, LEU2, CEN)	this study
VGY4317	BY background; MATa; Δpbn1::KAnMX; PBN1-MYC (VGc445, URA3, 2micron); GFP-PBN1TMD:LIV (VGc496, LEU2, CEN)	this study
VGY4318	BY background; MATa; Δpbn1::KAnMX; PBN1-MYC (VGc445, URA3, 2micron); GFP-PBN1Δ21-271 (VGc499, LEU2, CEN)	this study
VGY4320	BY background; MATa; Δpbn1::KAnMX; PBN1-MYC (VGc445, URA3, 2micron); GFP-PBN1Δ21-208Δ271-314 (VGc497, LEU2, CEN)	this study
VGY4321	BY background; MATa; Δpbn1::KAnMX; PBN1-MYC (VGc445, URA3, 2micron); GFP-PBN1Δ21-208Δ314-378 (VGc498, LEU2, CEN)	this study
VGY4326	BY background; MATa; Δpbn1::KAnMX; GFP-PBN1TMD:LIV (VGc496, LEU2, CEN)	this study
VGY4327	BY background; MATa; Δpbn1::KAnMX; GFP-PBN1TMD:LIV (VGc496, LEU2, CEN)	this study
VGY4353	BY background; MATx; CFTR-D208-HA (VGp332, URA3, CEN)	this study
VGY4362	BY background; MATx; GPI14-tdimer::KanMX; GFP-PBN1 (SWAT)	this study
VGY4368	BY background; MATa; GPI14-tdimer::HIS; TDH3P-KAR2ss-GFP-HDEL (VGp341, URA3, CEN)	this study
VGY4378	BY background; MATa; PBN1-GFP::KanMX; SEC63-mCherry (VGp241, URA3, CEN)	this study
VGY4379	BY background; MATx; Δpep4::KanMX; GFP-CPY*SRR_TMD (VGp468, URA3, CEN)	this study
VGY4383	BY background; MATa; ΔNg-CPY*FLAG (VGp381, URA3, CEN)	this study
VGY4384	BY background; MATa; PBN1-DAmP::KanMX; ΔNg-CPY*FLAG (VGp381, URA3, CEN)	this study
VGY4385	BY background; MATa; GPI14-DAmP::KanMX; ΔNg-CPY*FLAG (VGp381, URA3, CEN)	this study

VGY4402	BY background; MATx; Δpmt1::HIS; Δpmt2::KanMX; ΔNg-CPY*FLAG (VGp381, URA3, CEN)	this study
VGY4406	BY background; MATx; Δpmt4::KanMX; ΔNg-CPY*FLAG (VGp381, URA3, CEN)	this study
VGY4417	BY background; MATx; Δpmt1::HIS; Δpmt4::KanMX; ΔNg-CPY*FLAG (VGp381, URA3, CEN)	this study
VGY4418	BY background; MATx; Δpmt1::HIS; Δpmt5::KanMX; ΔNg-CPY*FLAG (VGp381, URA3, CEN)	this study
VGY4419	BY background; MATx; Δpmt2::HIS; Δpmt3::KanMX; ΔNg-CPY*FLAG (VGp381, URA3, CEN)	this study
VGY4433	BY background; MATx; PBN1-AID*-GFP::HYG; osTIR::HIS; ΔNg-CPY*FLAG (VGp381, URA3, CEN)	this study
VGY4434	BY background; MATa; Δpbn1::KAnMX; GFP-PBN1 (VGc476, LEU2, CEN), HA-CPY* (VGc421, URA3, CEN)	this study
VGY4438	BY background; MATa; Δpbn1::KAnMX; GFP-PBN1Δ21-208 (VGc489, LEU2, CEN), ΔNg-CPY*FLAG (VGp381, URA3, CEN)	this study
VGY4440	BY background; MATa; Δpbn1::KAnMX; GFP-PBN1TMD:LIV (VGc496, LEU2, CEN), HA-CPY* (VGc421, URA3, CEN)	this study
VGY4441	BY background; MATa; Δpbn1::KAnMX; GFP-PBN1TMD:LIV (VGc496, LEU2, CEN), ΔNg-CPY*FLAG (VGp381, URA3, CEN)	this study
VGY4454	BY background; MATx; PBN1-AID*-GFP::HYG; osTIR::HIS; HA-CPY* (VGc421, URA3, CEN)	this study
VGY4461	BY background; MATx; GPI14-AID*-GFP::HYG; osTIR::HIS	this study
VGY4464	BY background; MATx; GPI14-AID*-GFP::HYG; osTIR::HIS; ΔNg-CPY*FLAG (VGp381, URA3, CEN)	this study
VGY4473	BY background; MATa; Δpbn1::KAnMX; GFP-PBN1 (VGc476, LEU2, CEN), ΔNg-CPY*FLAG (VGp381, URA3, CEN)	this study
VGY4509	BY background; MATx; PBN1-AID*-GFP::HYG; osTIR::HIS; HA-GAS1*(VGp167, URA3, CEN)	this study
VGY4511	BY background; MATx; PBN1-AID*-GFP::HYG; osTIR::HIS; HA-GAS1wt (VGp310, URA3, CEN)	this study
VGY4512	BY background; MATx; PBN1-AID*-GFP::HYG; osTIR::HIS; GAS1P-HA-CPYwtSRR (VGc515, URA3, CEN)	this study
VGY4513	BY background; MATx; PBN1-AID*-GFP::HYG; osTIR::HIS; GAS1P-HA-CPY*SRR (VGc518, URA3, CEN)	this study
VGY4514	BY background; MATx; PBN1-AID*-GFP::HYG; osTIR::HIS; HA-GAS1*TMD (VGp176, URA3, CEN)	this study
VGY4517	BY background; MATa; GAS1P-HA-CPY*SRR (VGc518, URA3, CEN)	this study
VGY4518	BY background; MATa; GAL1P-3HA-PBN1::NAT; GAS1P-HA-CPY*SRR (VGc518, URA3, CEN)	this study
VGY4520	BY background; MATa; PBN1-DAmP::KanMX; GAS1P-HA-CPY*SRR (VGc518, URA3, CEN)	this study

Appendix Table S2. List of plasmids used in this study.

EP = endogenous promoter

Plasmid	Backbone	Description	Reference
VGp161	pRS316	GAL4P-LEU2-TMD-Gas1*	this study
VGp165	pRS306	EP-HA-GAS1*	PMID: 16319176
VGp167	pRS316	EP-HA-GAS1*	PMID: 21147851
VGp176	pRS316	EP-HA-GAS1*TMD	PMID: 27325793
VGp241	pRS316	SEC63-mCherry	this study
VGp242	pRS315	SEC63-mCherry	this study
VGp310	pRS316	EP-HA-GAS1	PMID: 21147851
VGp332	pRS316	Δ208-CFTR-HA	PMID: 15340068
VGp341	pRS316	TDH3P-KARss-GFP-HDEL	this study
VGp381	pRS316	ΔNg-CPY*FLAG	PMID: 15769847
VGp468	pRS316	GAS1P-GFP-CPY*SRR_TMD	this study
VGc226	pRS306	EP-GFP-GAS1*	this study
VGc418	pRS316	GAS1P-HA-CPY*SRR_TMD	this study
VGc421	pRS316	GAS1P-HA-CPY*	this study
VGc445	pESC-URA	EP-PBN1-myc	this study
VGc476	pRS315	EP-GFP-PBN1	this study
VGc489	pRS315	EP-GFP-PBN1Δ21-208	this study
VGc490	pRS315	EP-GFP-PBN1ΔTMD	this study
VGc491	pRS315	EP-GFP-PBN1ΔTMD	this study
VGc496	pRS315	EP-GFP-PBN1TMD:LIV	this study
VGc497	pRS315	EP-GFP-PBN1Δ21-208Δ271-314	this study
VGc498	pRS315	EP-GFP-PBN1Δ21-208Δ314-378	this study
VGc499	pRS315	EP-GFP-PBN1Δ21-271	this study
VGc515	pRS316	GAS1P-HA-CPYwtSRR	this study
VGc518	pRS316	GAS1P-HA-CPY*SRR	this study
