# Supplementary figures and images for: O-mannosylation of misfolded ER proteins promotes ERAD
Source: EMBO J. 2025 Dec 5;45(2):564–91. doi: 10.1038/s44318-025-00647-2 (PMC12811338; doi:10.1038/s44318-025-00647-2)

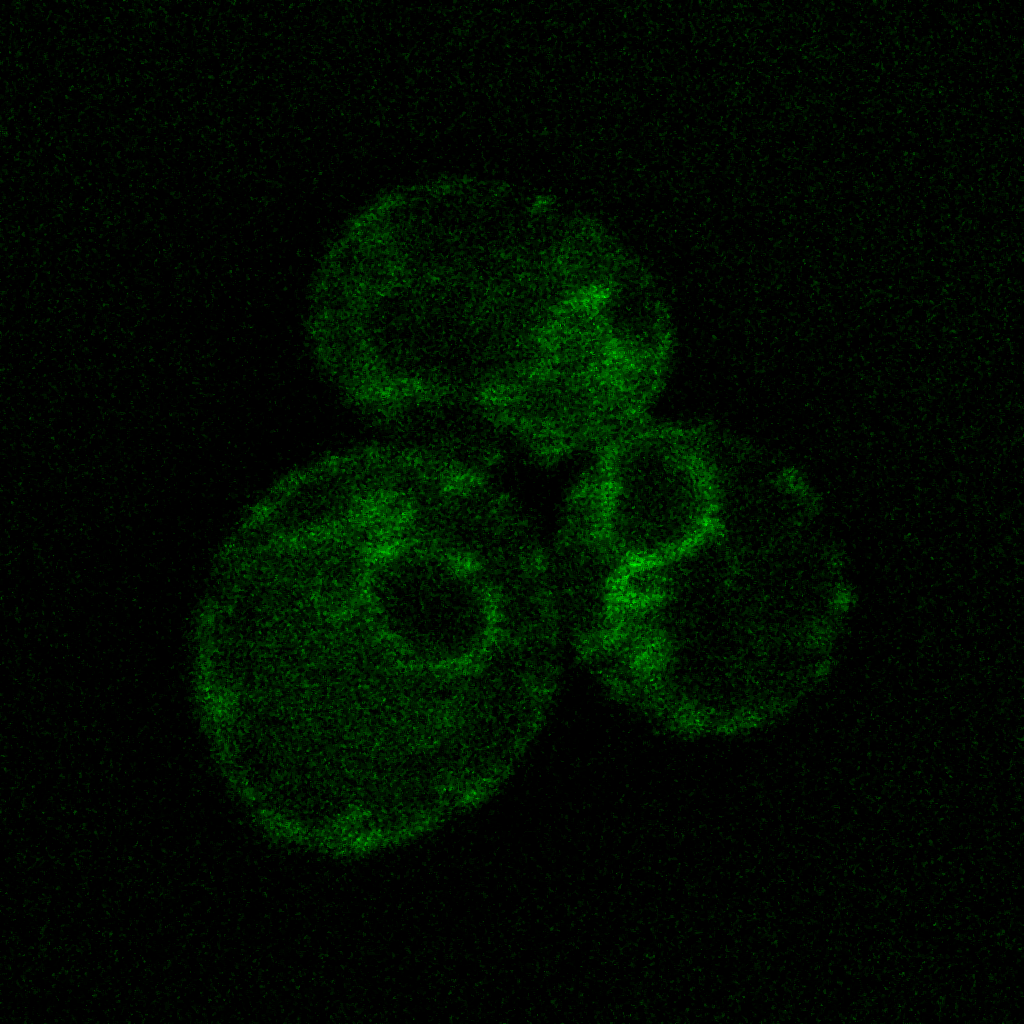

Supplement: Supplementary file 4 — Source data Fig. 2 [file 44318_2025_647_MOESM4_ESM.zip › SD figure 2/2B ch_00.tif]

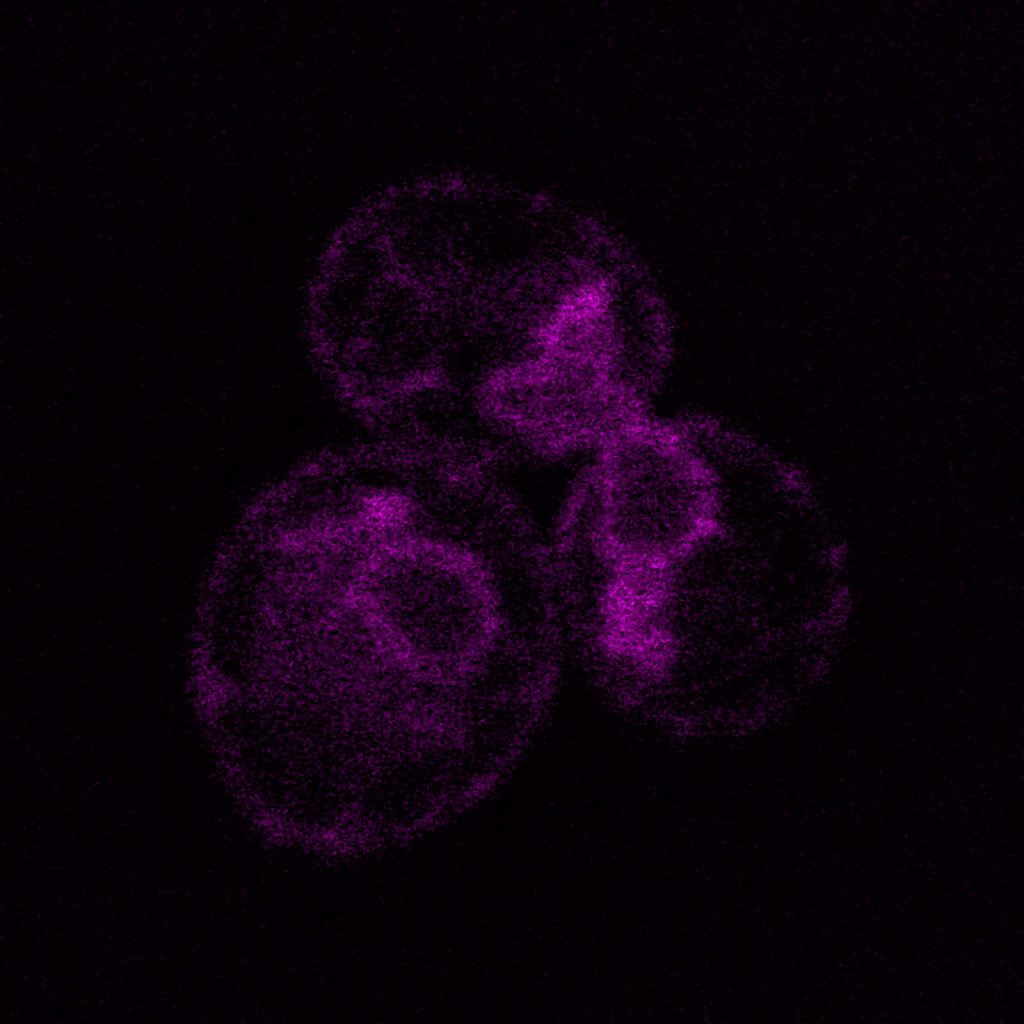

Supplement: Supplementary file 4 — Source data Fig. 2 [file 44318_2025_647_MOESM4_ESM.zip › SD figure 2/2B ch_01.tif]

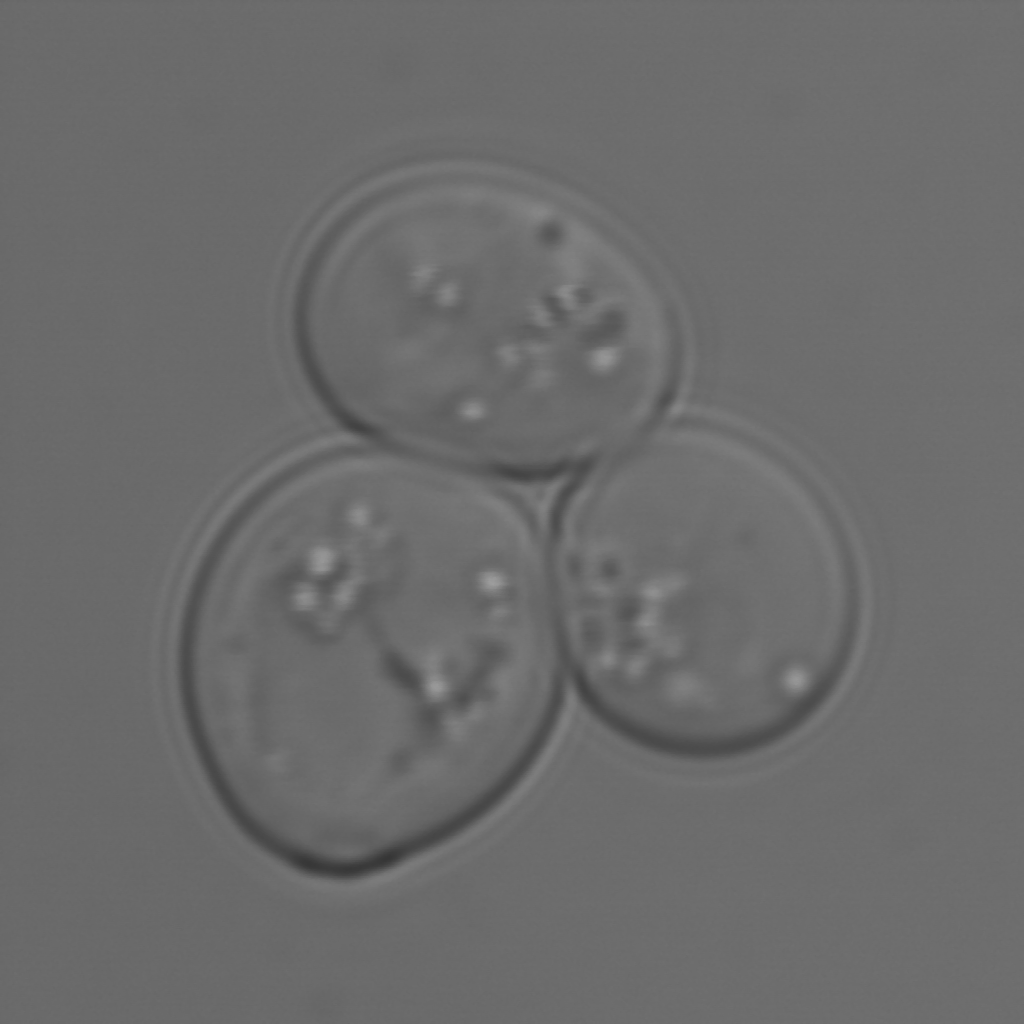

Supplement: Supplementary file 4 — Source data Fig. 2 [file 44318_2025_647_MOESM4_ESM.zip › SD figure 2/2B ch_02.tif]

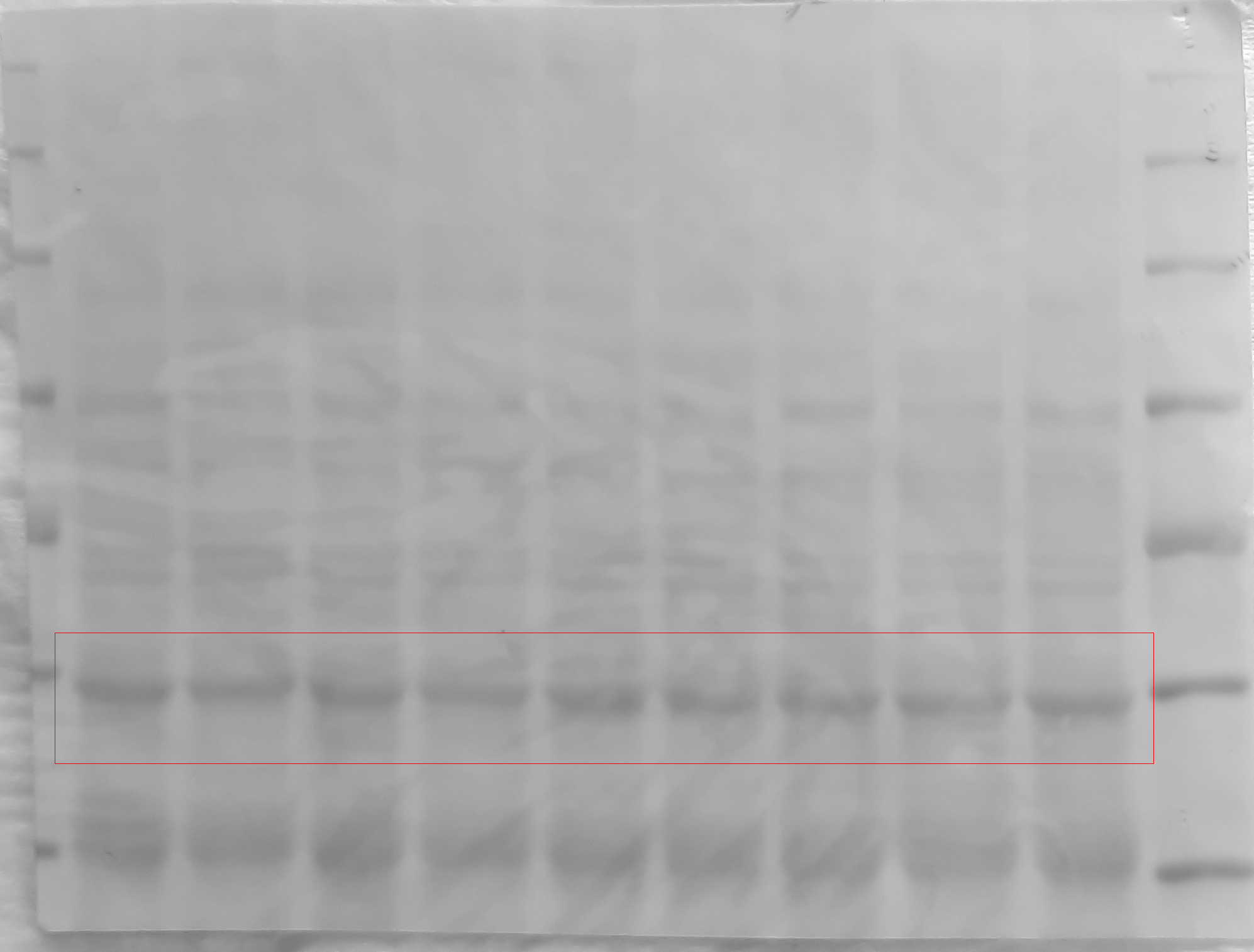

Supplement: Supplementary file 4 — Source data Fig. 2 [file 44318_2025_647_MOESM4_ESM.zip › SD figure 2/2C Ponceau.tif]

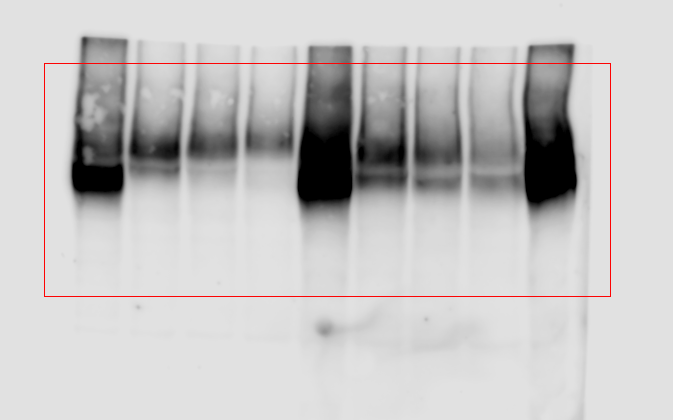

Supplement: Supplementary file 4 — Source data Fig. 2 [file 44318_2025_647_MOESM4_ESM.zip › SD figure 2/2C WB.tif]

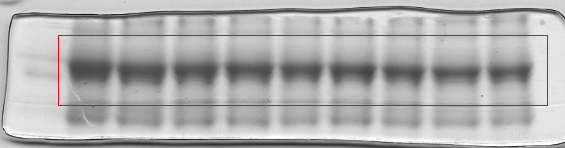

Supplement: Supplementary file 4 — Source data Fig. 2 [file 44318_2025_647_MOESM4_ESM.zip › SD figure 2/2E Coomassie.tif]

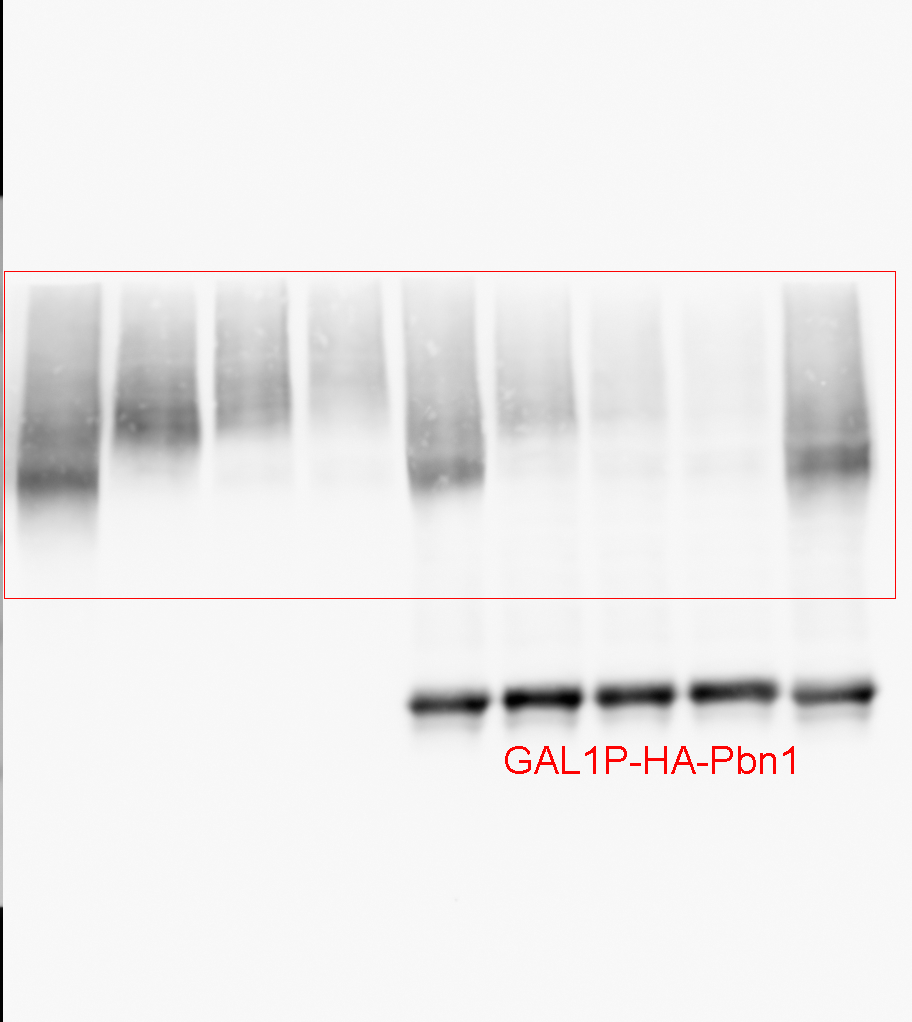

Supplement: Supplementary file 4 — Source data Fig. 2 [file 44318_2025_647_MOESM4_ESM.zip › SD figure 2/2E WB.tif]

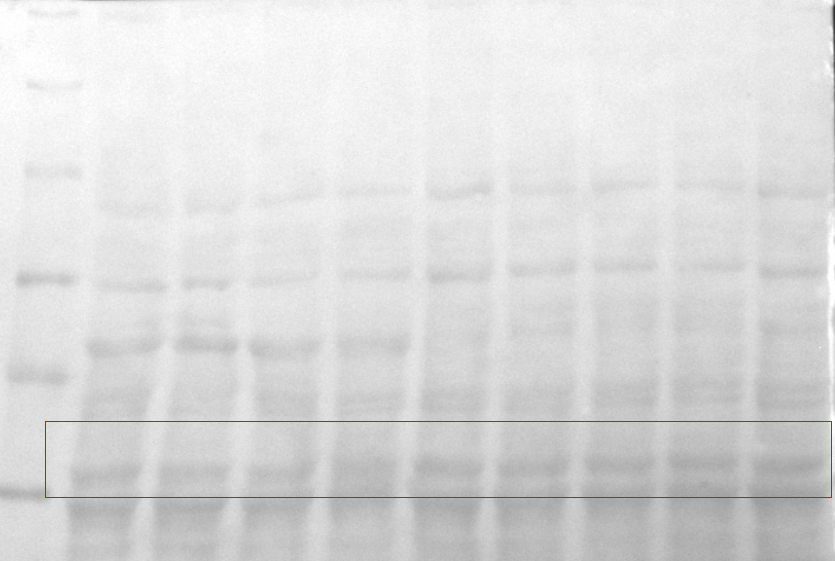

Supplement: Supplementary file 4 — Source data Fig. 2 [file 44318_2025_647_MOESM4_ESM.zip › SD figure 2/2G Ponceau.tif]

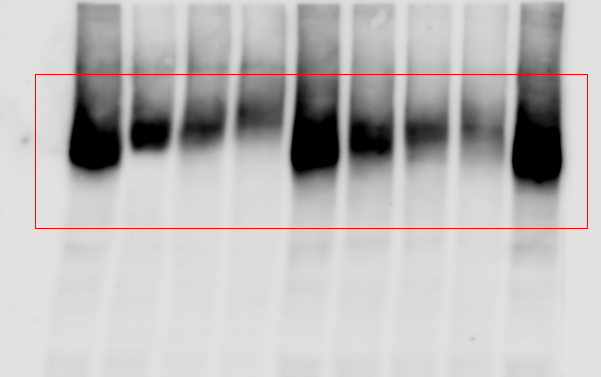

Supplement: Supplementary file 4 — Source data Fig. 2 [file 44318_2025_647_MOESM4_ESM.zip › SD figure 2/2G WB.tif]

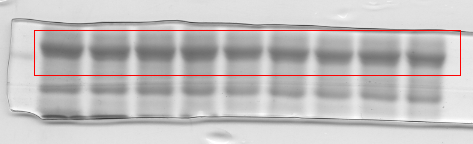

Supplement: Supplementary file 4 — Source data Fig. 2 [file 44318_2025_647_MOESM4_ESM.zip › SD figure 2/2I Commassie.tif]

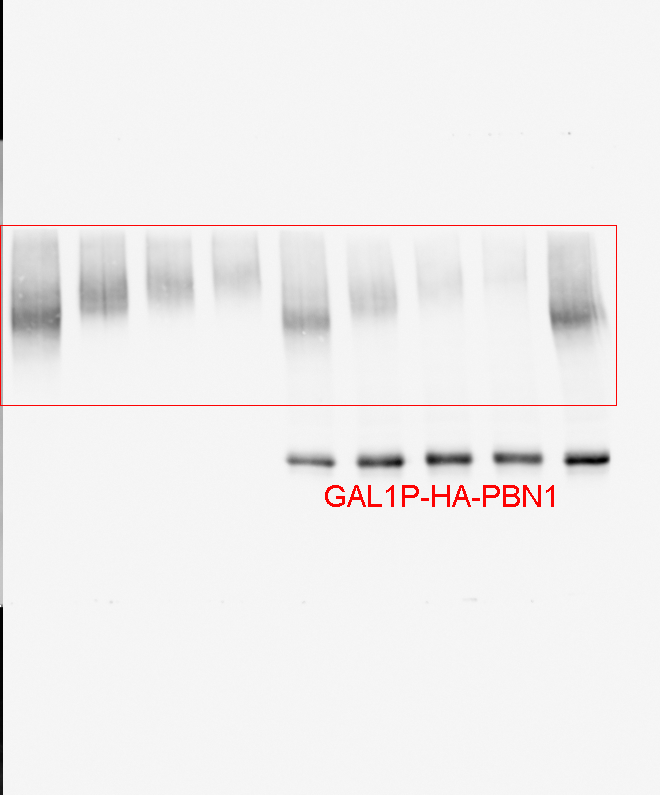

Supplement: Supplementary file 4 — Source data Fig. 2 [file 44318_2025_647_MOESM4_ESM.zip › SD figure 2/2I WB.tif]

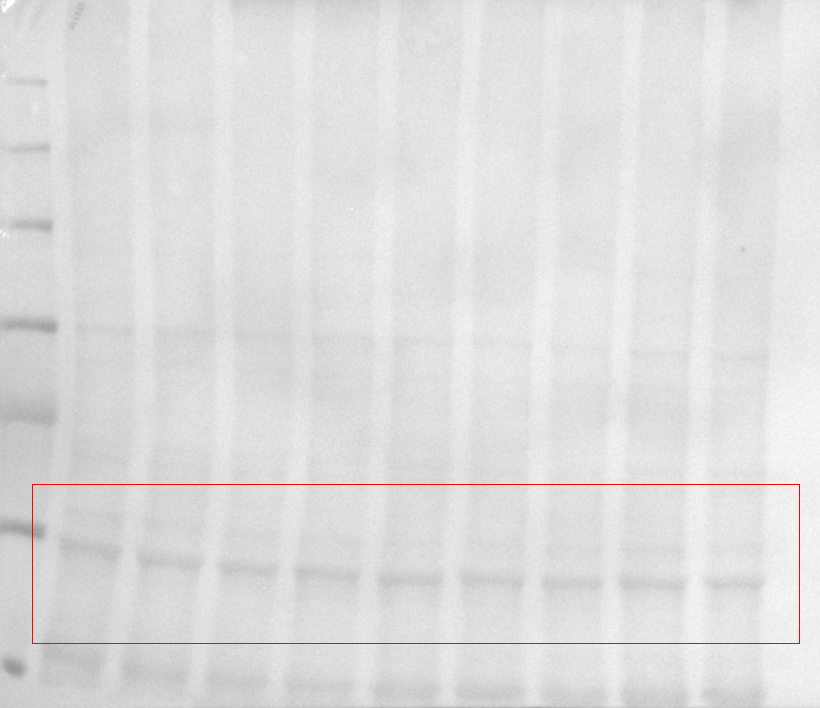

Supplement: Supplementary file 4 — Source data Fig. 2 [file 44318_2025_647_MOESM4_ESM.zip › SD figure 2/2K Ponceau.tif]

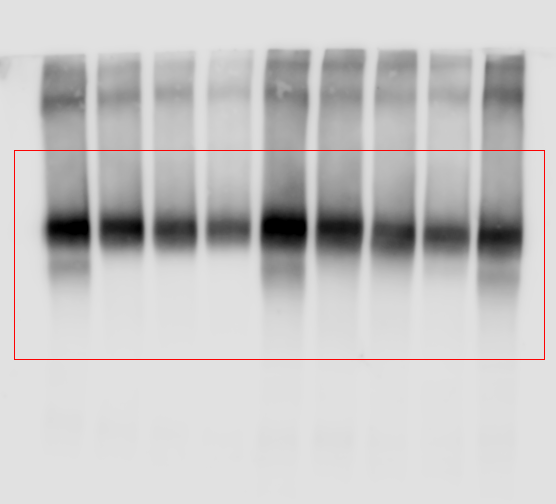

Supplement: Supplementary file 4 — Source data Fig. 2 [file 44318_2025_647_MOESM4_ESM.zip › SD figure 2/2K WB.tif]

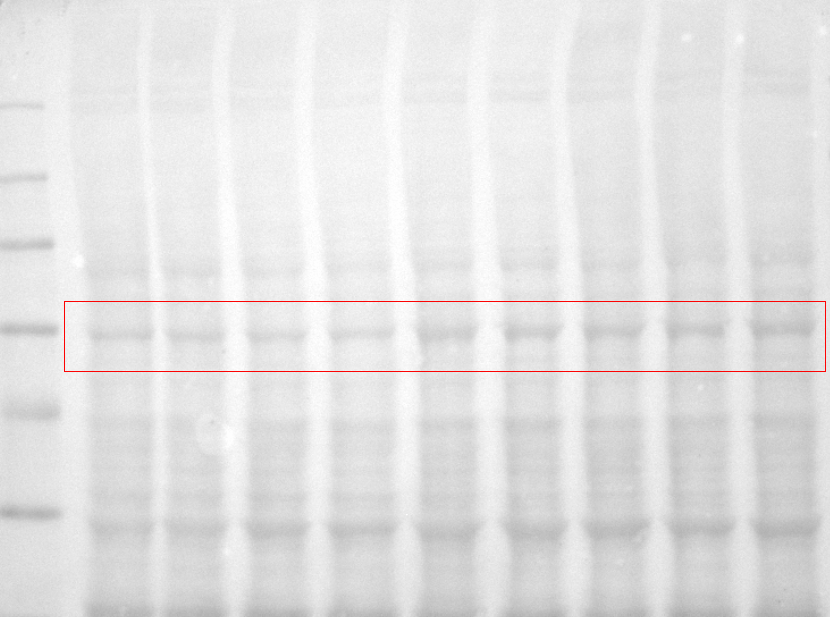

Supplement: Supplementary file 4 — Source data Fig. 2 [file 44318_2025_647_MOESM4_ESM.zip › SD figure 2/2M Ponceau.tif]

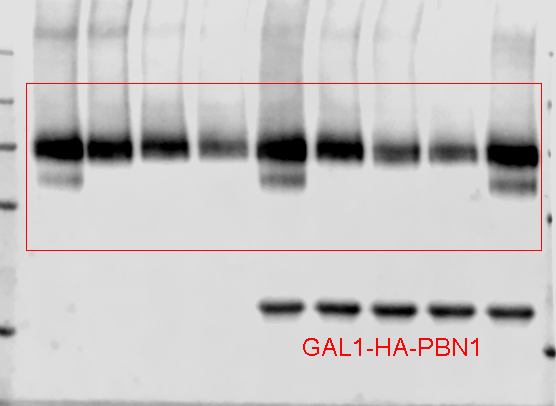

Supplement: Supplementary file 4 — Source data Fig. 2 [file 44318_2025_647_MOESM4_ESM.zip › SD figure 2/2M WB.tif]

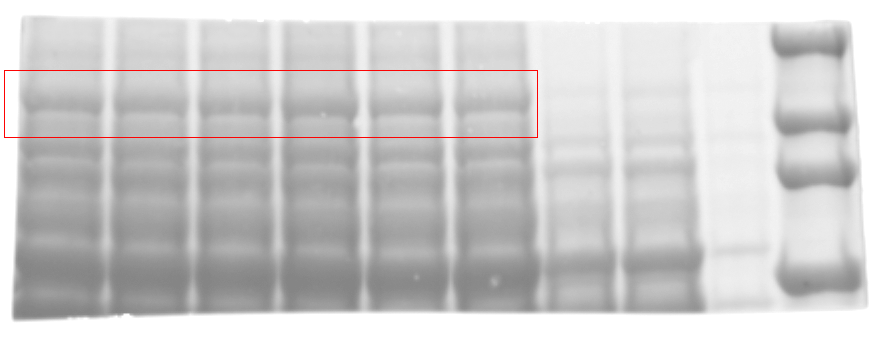

Supplement: Supplementary file 4 — Source data Fig. 2 [file 44318_2025_647_MOESM4_ESM.zip › SD figure 2/2O Ponceau.tif]

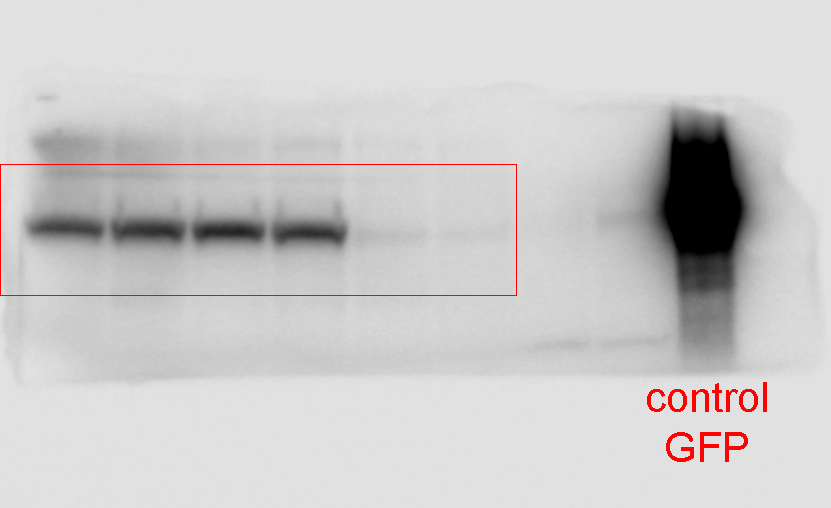

Supplement: Supplementary file 4 — Source data Fig. 2 [file 44318_2025_647_MOESM4_ESM.zip › SD figure 2/2O WB.tif]

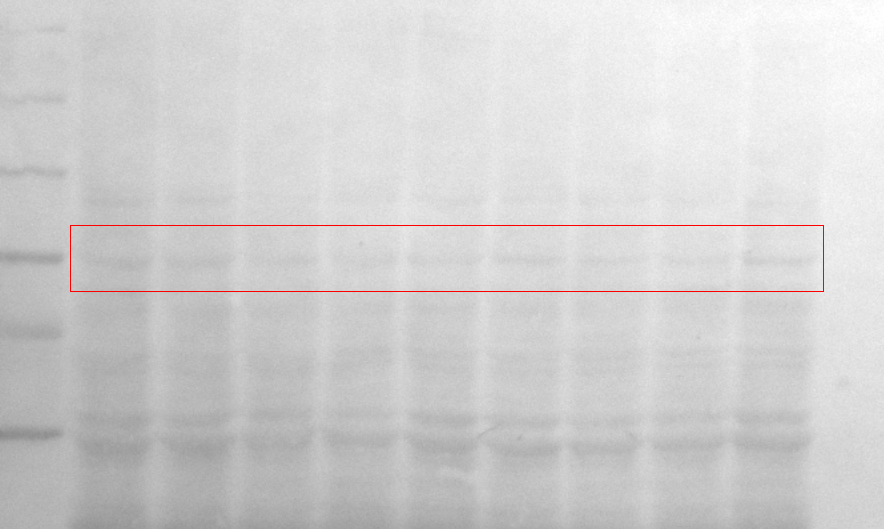

Supplement: Supplementary file 4 — Source data Fig. 2 [file 44318_2025_647_MOESM4_ESM.zip › SD figure 2/2P Ponceau.tif]

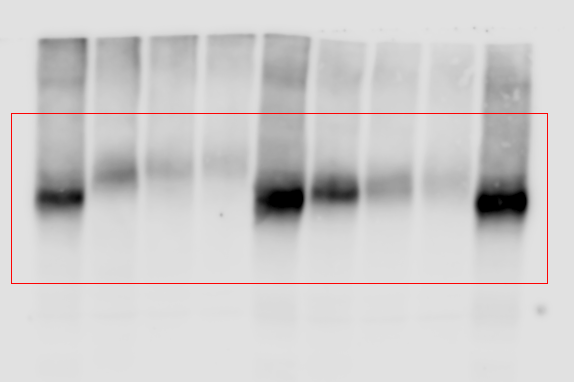

Supplement: Supplementary file 4 — Source data Fig. 2 [file 44318_2025_647_MOESM4_ESM.zip › SD figure 2/2P WB.tif]

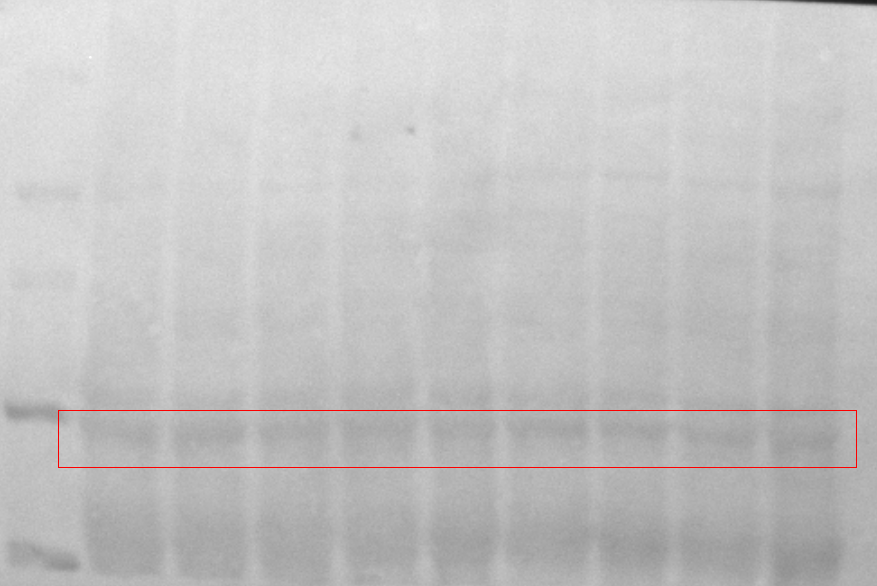

Supplement: Supplementary file 4 — Source data Fig. 2 [file 44318_2025_647_MOESM4_ESM.zip › SD figure 2/2R Ponceau.tif]

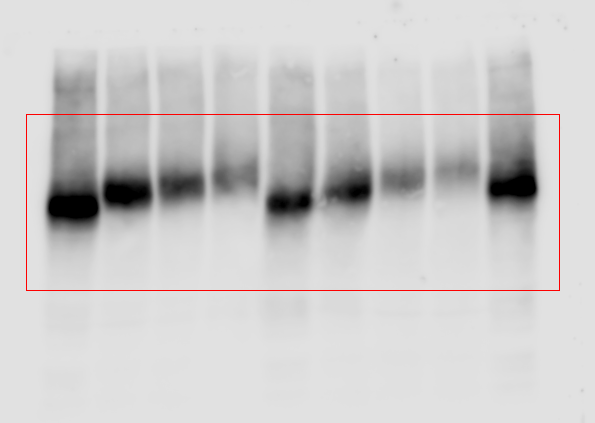

Supplement: Supplementary file 4 — Source data Fig. 2 [file 44318_2025_647_MOESM4_ESM.zip › SD figure 2/2R WB.tif]

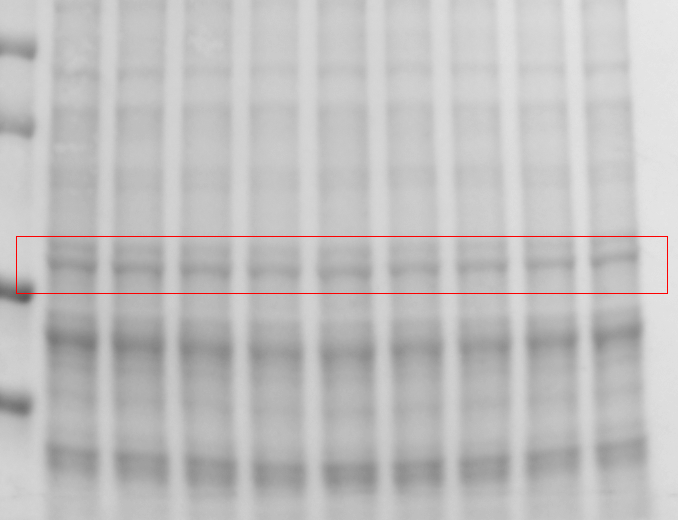

Supplement: Supplementary file 4 — Source data Fig. 2 [file 44318_2025_647_MOESM4_ESM.zip › SD figure 2/2T Ponceau.tif]

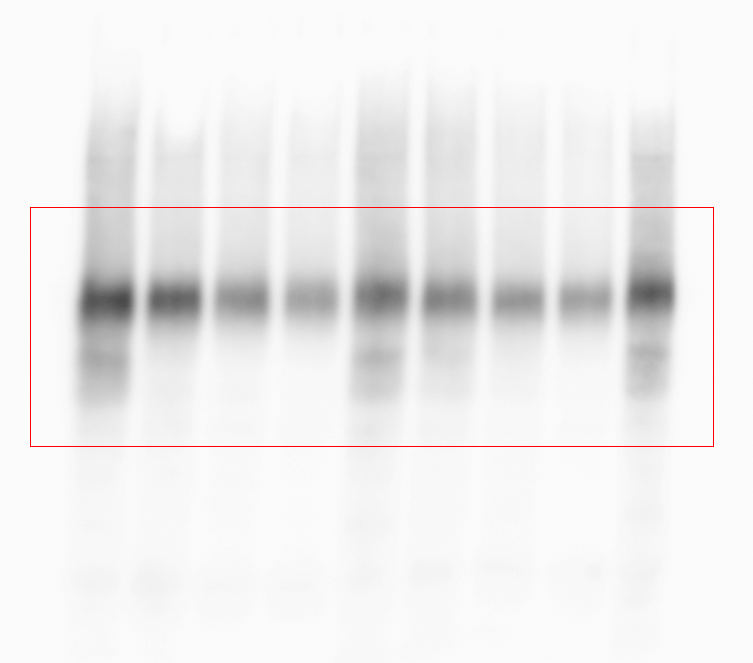

Supplement: Supplementary file 4 — Source data Fig. 2 [file 44318_2025_647_MOESM4_ESM.zip › SD figure 2/2T WB.tif]

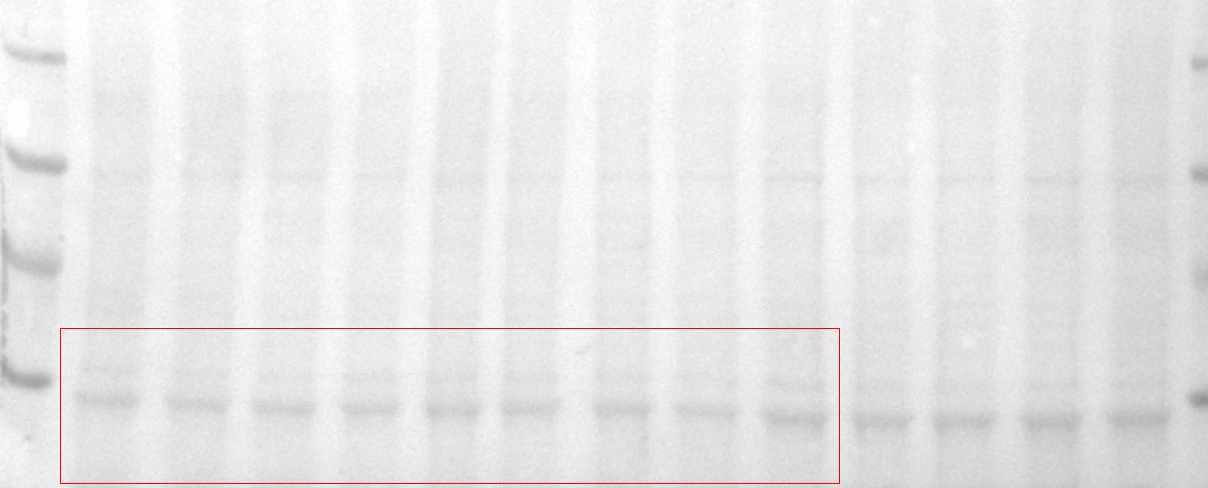

Supplement: Supplementary file 5 — Source data Fig. 3 [file 44318_2025_647_MOESM5_ESM.zip › SD figure 3/3A Ponceau.tif]

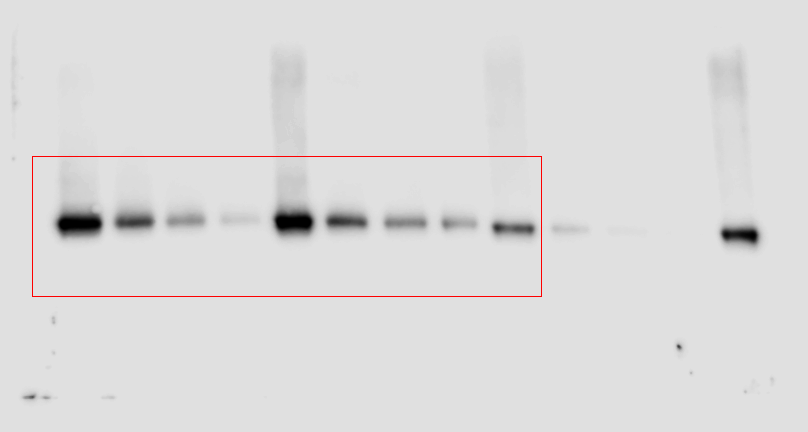

Supplement: Supplementary file 5 — Source data Fig. 3 [file 44318_2025_647_MOESM5_ESM.zip › SD figure 3/3A WB.tif]

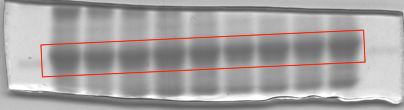

Supplement: Supplementary file 5 — Source data Fig. 3 [file 44318_2025_647_MOESM5_ESM.zip › SD figure 3/3C Coomassie.tif]

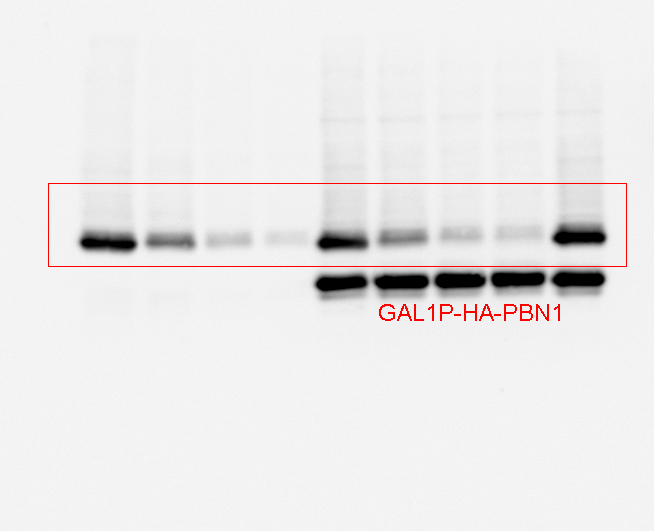

Supplement: Supplementary file 5 — Source data Fig. 3 [file 44318_2025_647_MOESM5_ESM.zip › SD figure 3/3C WB.tif]

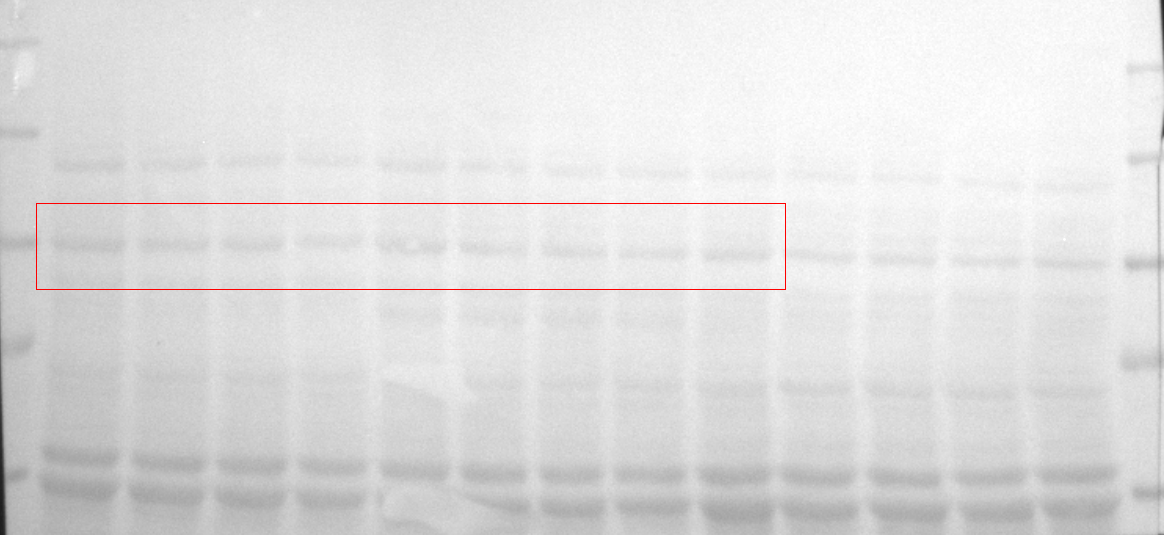

Supplement: Supplementary file 5 — Source data Fig. 3 [file 44318_2025_647_MOESM5_ESM.zip › SD figure 3/3F Ponceau.tif]

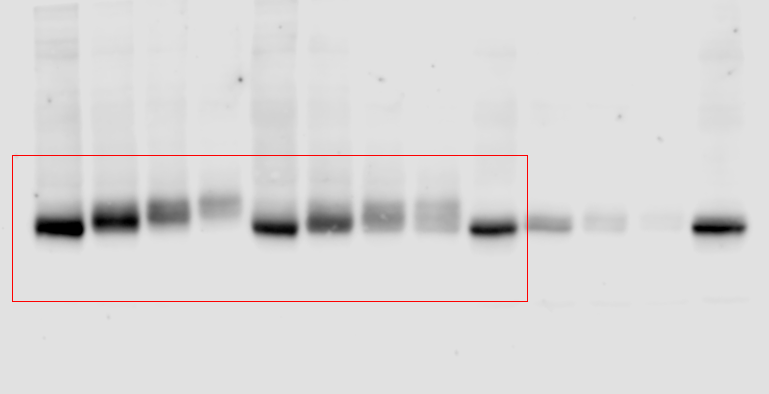

Supplement: Supplementary file 5 — Source data Fig. 3 [file 44318_2025_647_MOESM5_ESM.zip › SD figure 3/3F WB.tif]

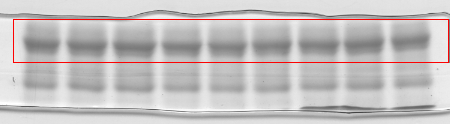

Supplement: Supplementary file 5 — Source data Fig. 3 [file 44318_2025_647_MOESM5_ESM.zip › SD figure 3/3H Coomassie.tif]

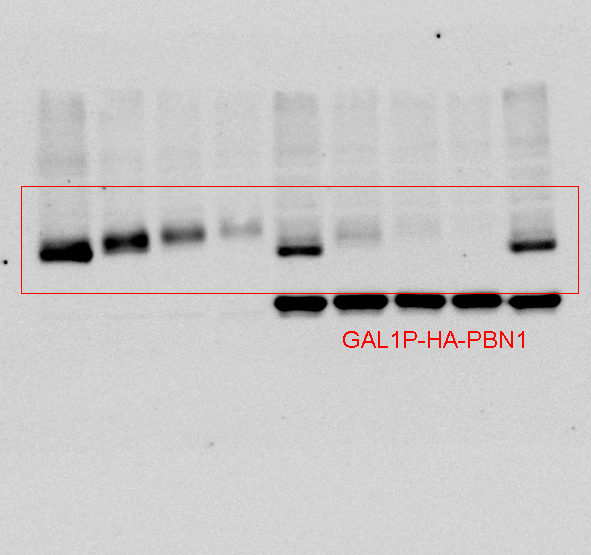

Supplement: Supplementary file 5 — Source data Fig. 3 [file 44318_2025_647_MOESM5_ESM.zip › SD figure 3/3H WB.tif]

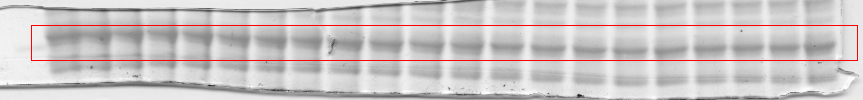

Supplement: Supplementary file 5 — Source data Fig. 3 [file 44318_2025_647_MOESM5_ESM.zip › SD figure 3/3J Coomassie.tif]

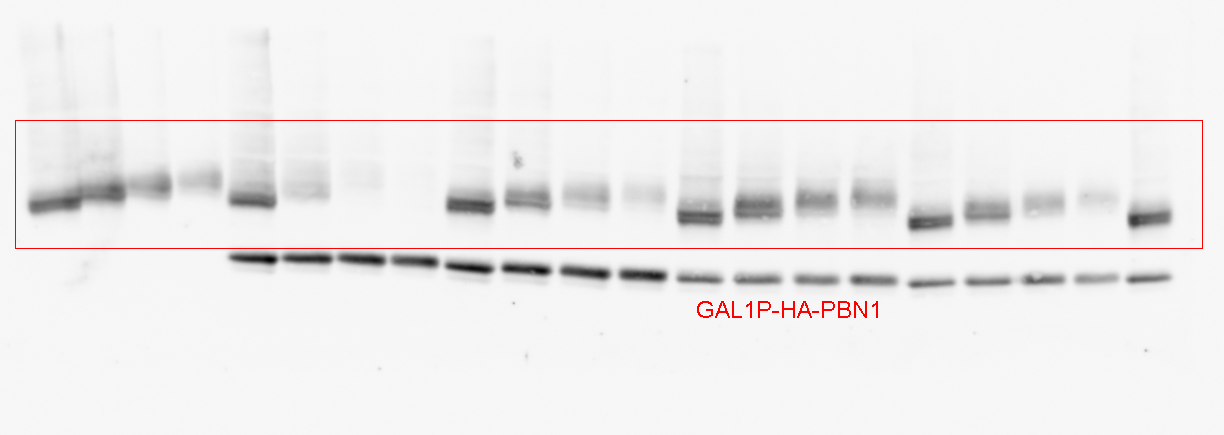

Supplement: Supplementary file 5 — Source data Fig. 3 [file 44318_2025_647_MOESM5_ESM.zip › SD figure 3/3J WB.tif]

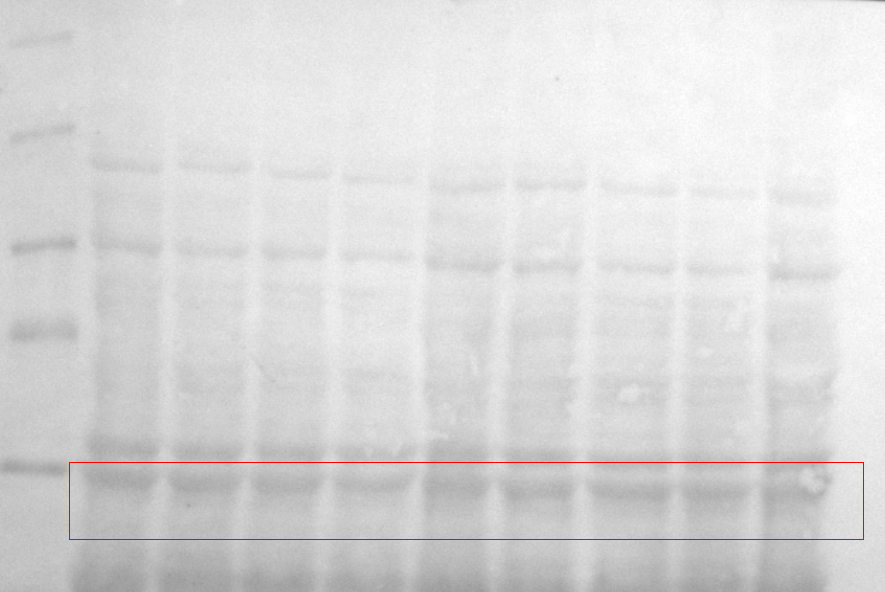

Supplement: Supplementary file 5 — Source data Fig. 3 [file 44318_2025_647_MOESM5_ESM.zip › SD figure 3/3L Ponceau.tif]

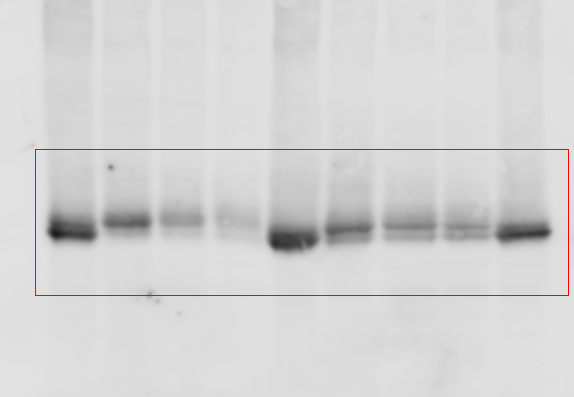

Supplement: Supplementary file 5 — Source data Fig. 3 [file 44318_2025_647_MOESM5_ESM.zip › SD figure 3/3L WB.tif]

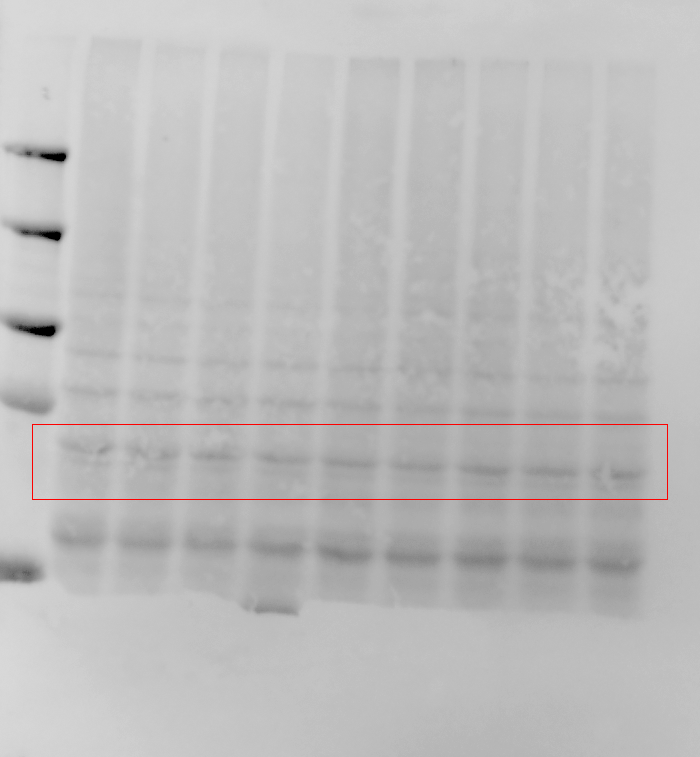

Supplement: Supplementary file 5 — Source data Fig. 3 [file 44318_2025_647_MOESM5_ESM.zip › SD figure 3/3N Ponceau.tif]

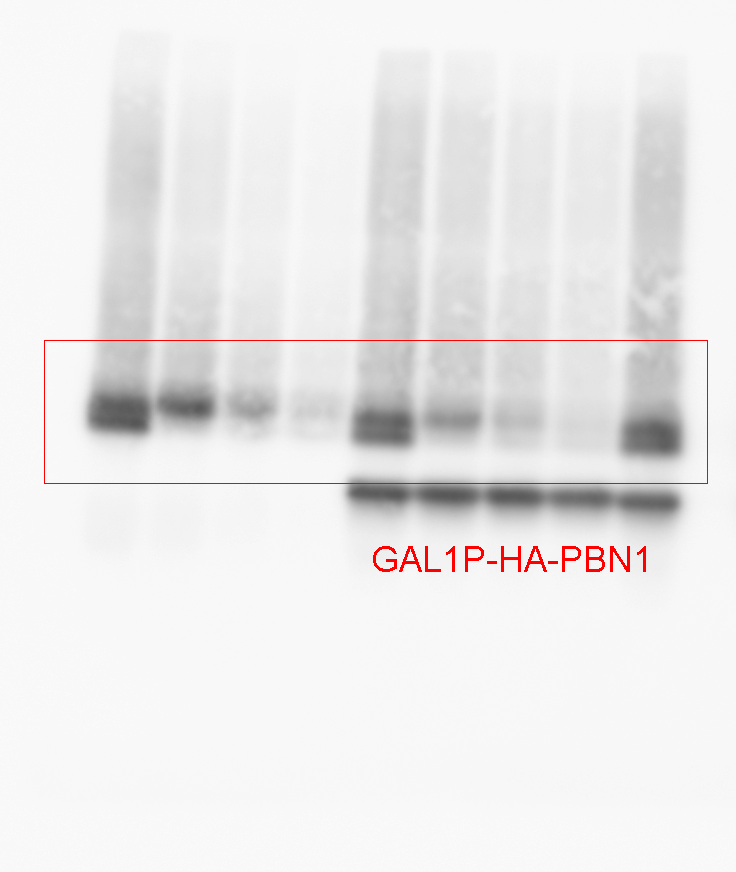

Supplement: Supplementary file 5 — Source data Fig. 3 [file 44318_2025_647_MOESM5_ESM.zip › SD figure 3/3N WB.tif]

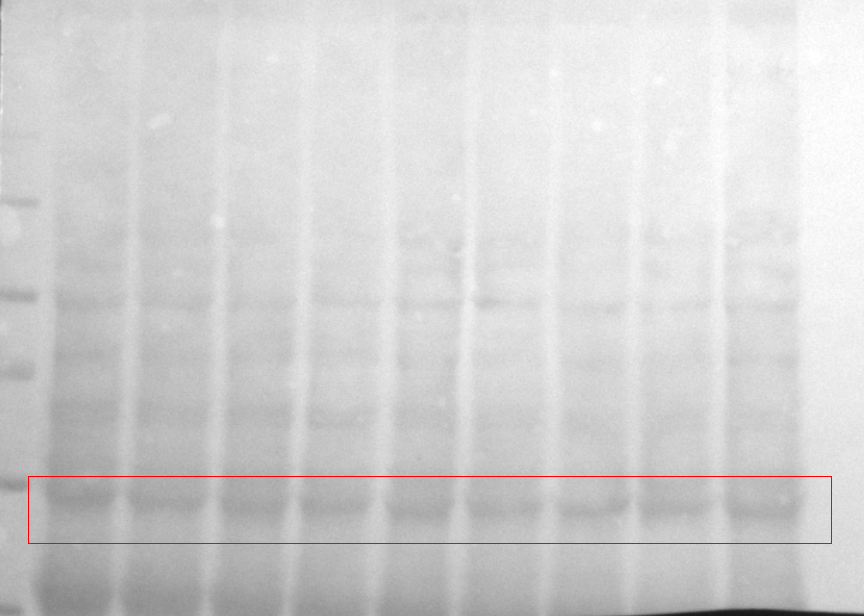

Supplement: Supplementary file 5 — Source data Fig. 3 [file 44318_2025_647_MOESM5_ESM.zip › SD figure 3/3P Ponceau.tif]

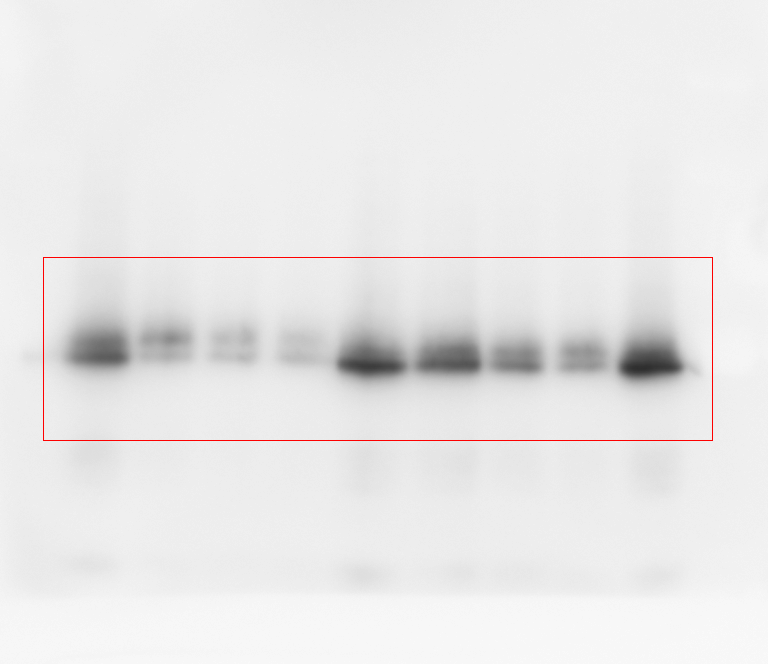

Supplement: Supplementary file 5 — Source data Fig. 3 [file 44318_2025_647_MOESM5_ESM.zip › SD figure 3/3P WB.tif]

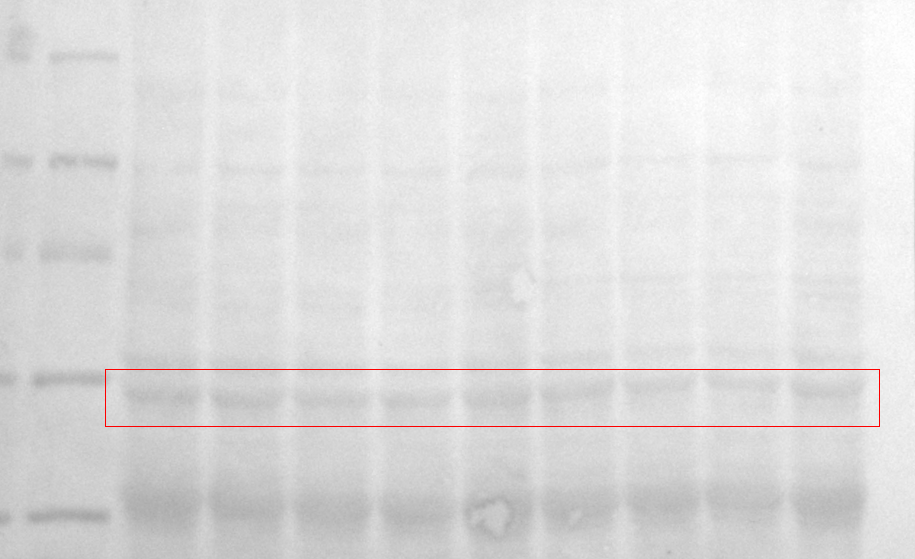

Supplement: Supplementary file 5 — Source data Fig. 3 [file 44318_2025_647_MOESM5_ESM.zip › SD figure 3/3R Ponceau.tif]

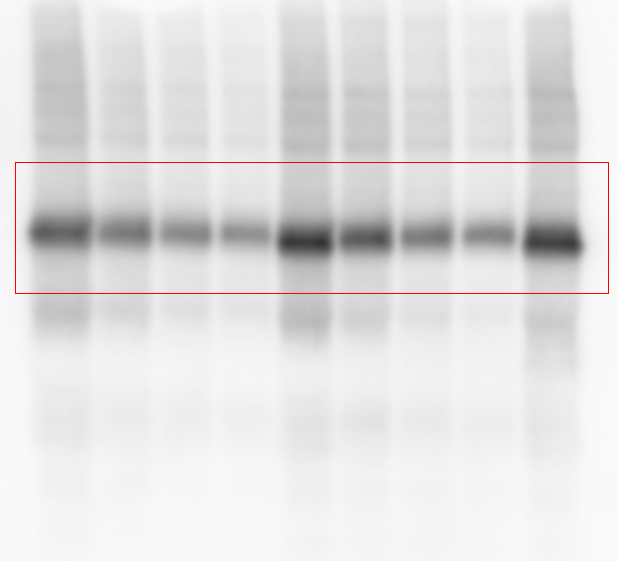

Supplement: Supplementary file 5 — Source data Fig. 3 [file 44318_2025_647_MOESM5_ESM.zip › SD figure 3/3R WB.tif]

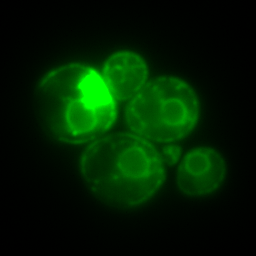

Supplement: Supplementary file 6 — Source data Fig. 4 [file 44318_2025_647_MOESM6_ESM.zip › SD figure 4/4A ch00.tif]

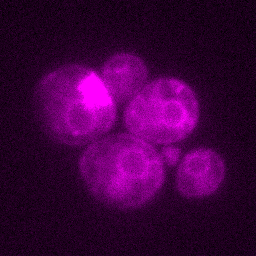

Supplement: Supplementary file 6 — Source data Fig. 4 [file 44318_2025_647_MOESM6_ESM.zip › SD figure 4/4A ch01.tif]

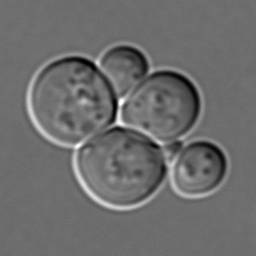

Supplement: Supplementary file 6 — Source data Fig. 4 [file 44318_2025_647_MOESM6_ESM.zip › SD figure 4/4A ch02.tif]

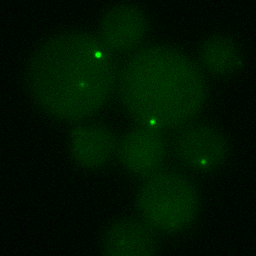

Supplement: Supplementary file 6 — Source data Fig. 4 [file 44318_2025_647_MOESM6_ESM.zip › SD figure 4/4B ch00.tif]

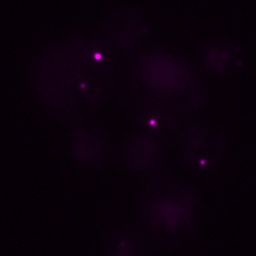

Supplement: Supplementary file 6 — Source data Fig. 4 [file 44318_2025_647_MOESM6_ESM.zip › SD figure 4/4B ch01.tif]

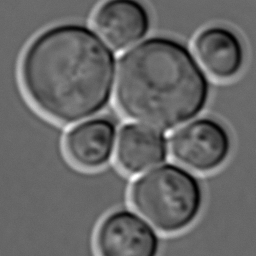

Supplement: Supplementary file 6 — Source data Fig. 4 [file 44318_2025_647_MOESM6_ESM.zip › SD figure 4/4B ch02.tif]

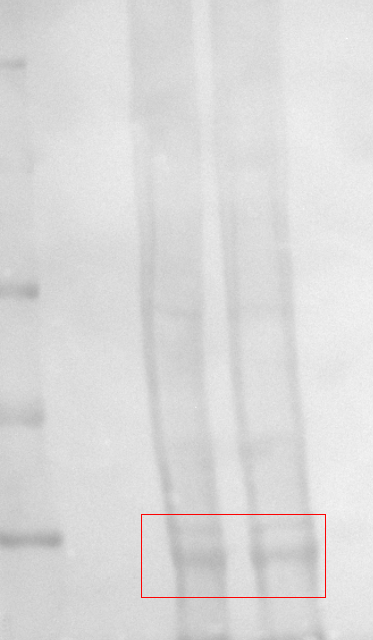

Supplement: Supplementary file 7 — Source data Fig. 5 [file 44318_2025_647_MOESM7_ESM.zip › SD figure 5/5B Ponceau.tif]

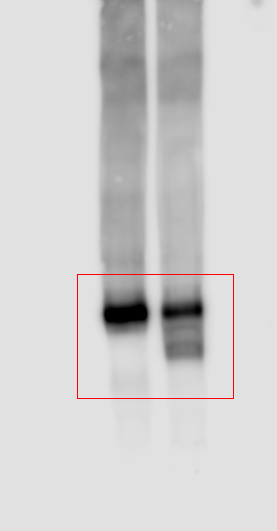

Supplement: Supplementary file 7 — Source data Fig. 5 [file 44318_2025_647_MOESM7_ESM.zip › SD figure 5/5B WB.tif]

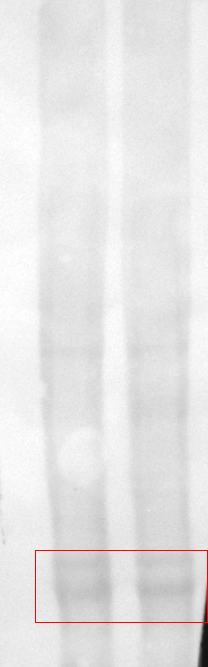

Supplement: Supplementary file 7 — Source data Fig. 5 [file 44318_2025_647_MOESM7_ESM.zip › SD figure 5/5C Ponceau.tif]

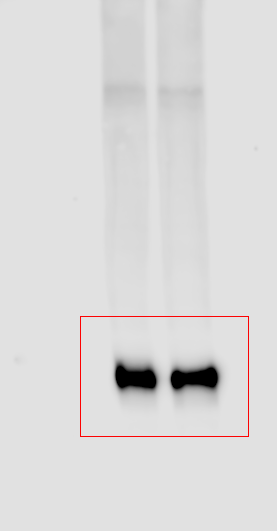

Supplement: Supplementary file 7 — Source data Fig. 5 [file 44318_2025_647_MOESM7_ESM.zip › SD figure 5/5C WB.tif]

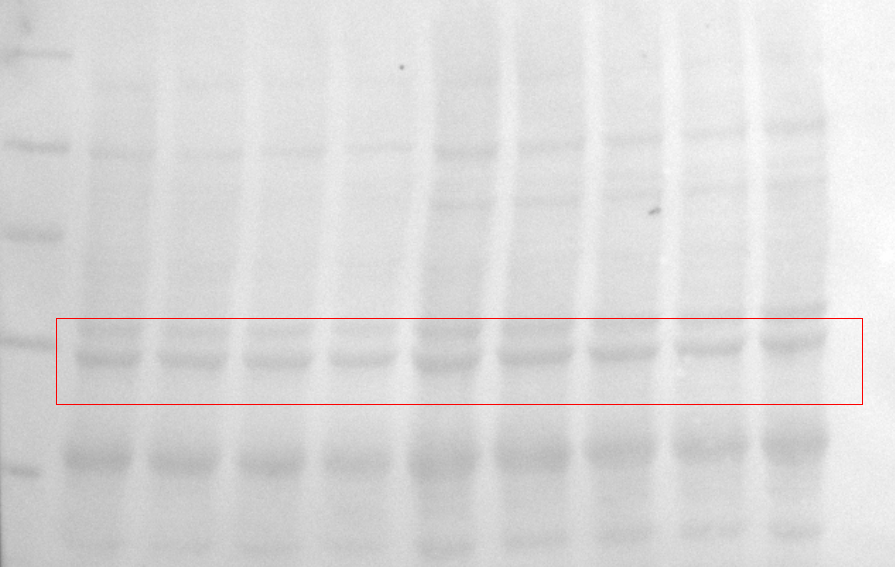

Supplement: Supplementary file 7 — Source data Fig. 5 [file 44318_2025_647_MOESM7_ESM.zip › SD figure 5/5D Ponceau.tif]

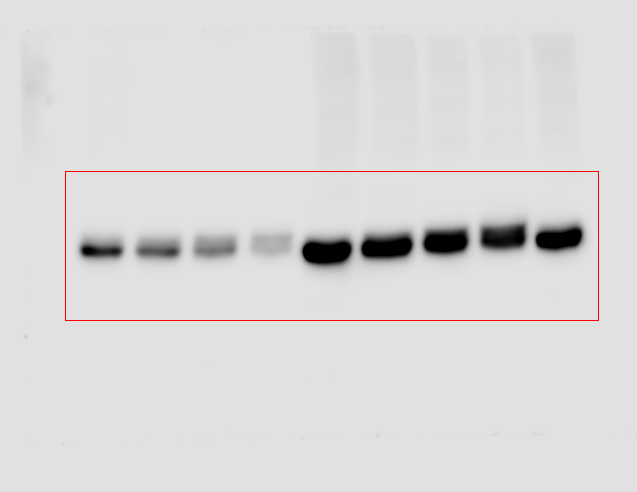

Supplement: Supplementary file 7 — Source data Fig. 5 [file 44318_2025_647_MOESM7_ESM.zip › SD figure 5/5D WB.tif]

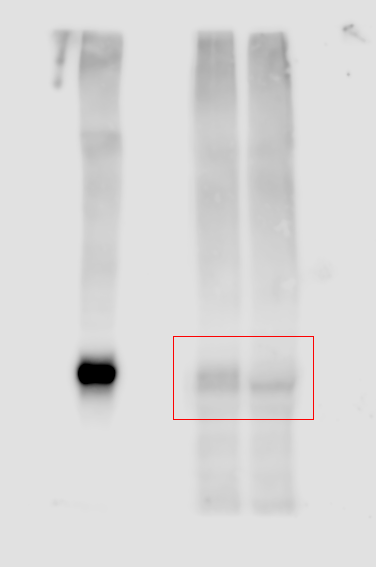

Supplement: Supplementary file 7 — Source data Fig. 5 [file 44318_2025_647_MOESM7_ESM.zip › SD figure 5/5F WB.tif]

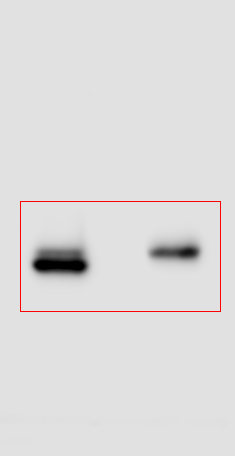

Supplement: Supplementary file 7 — Source data Fig. 5 [file 44318_2025_647_MOESM7_ESM.zip › SD figure 5/5G WB control.tif]

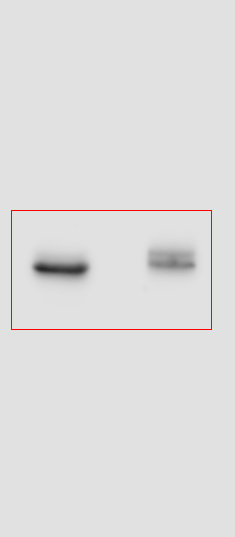

Supplement: Supplementary file 7 — Source data Fig. 5 [file 44318_2025_647_MOESM7_ESM.zip › SD figure 5/5G WB hrd1.tif]

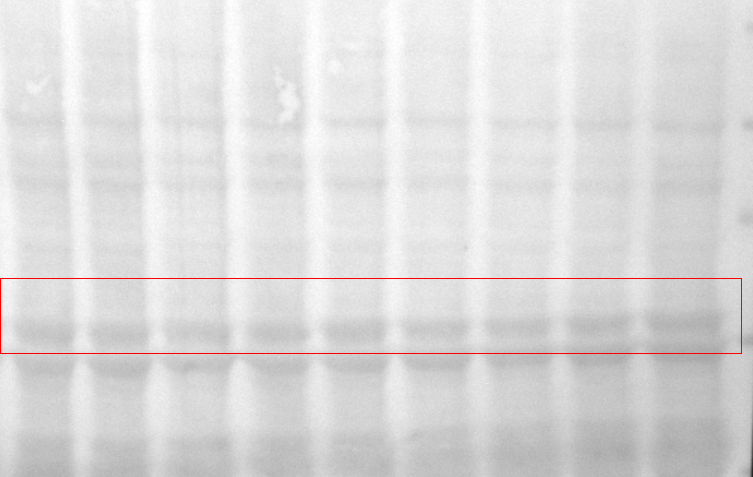

Supplement: Supplementary file 7 — Source data Fig. 5 [file 44318_2025_647_MOESM7_ESM.zip › SD figure 5/5H Ponceau.tif]

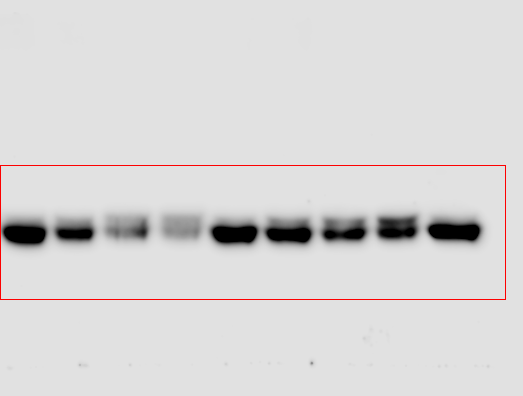

Supplement: Supplementary file 7 — Source data Fig. 5 [file 44318_2025_647_MOESM7_ESM.zip › SD figure 5/5H WB.tif]

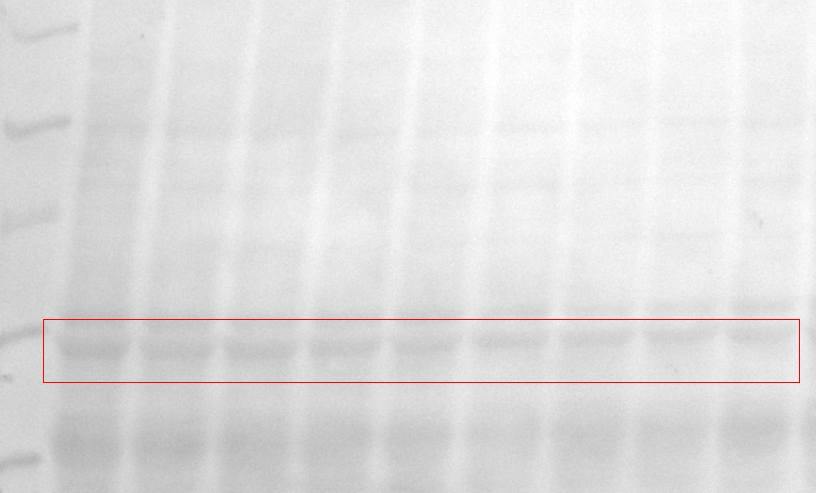

Supplement: Supplementary file 7 — Source data Fig. 5 [file 44318_2025_647_MOESM7_ESM.zip › SD figure 5/5J Ponceau.tif]

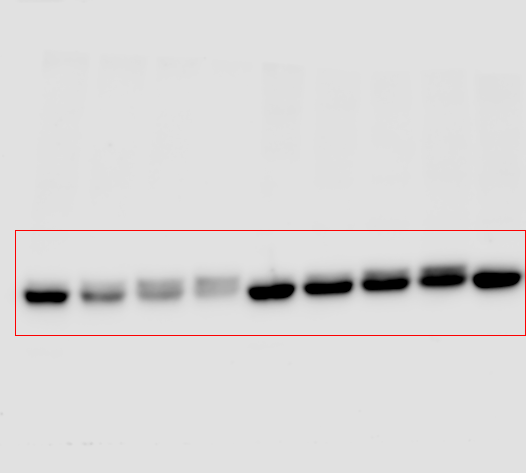

Supplement: Supplementary file 7 — Source data Fig. 5 [file 44318_2025_647_MOESM7_ESM.zip › SD figure 5/5J WB.tif]

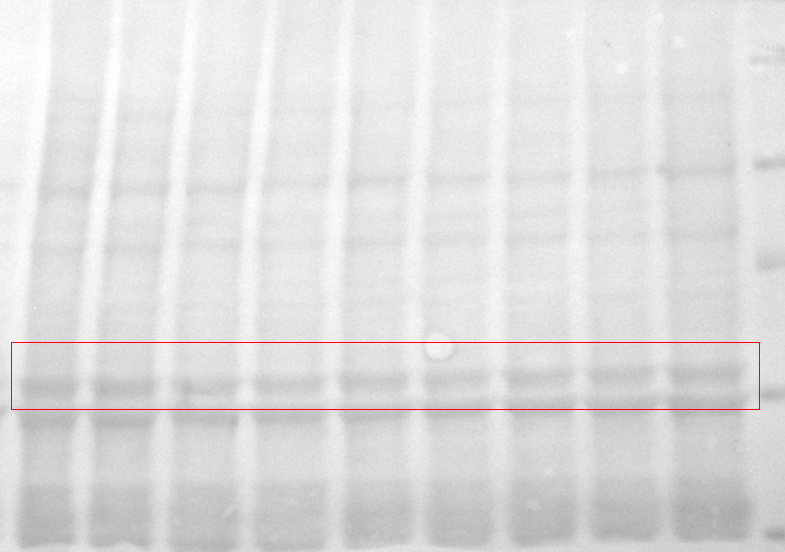

Supplement: Supplementary file 7 — Source data Fig. 5 [file 44318_2025_647_MOESM7_ESM.zip › SD figure 5/5L Ponceau.tif]

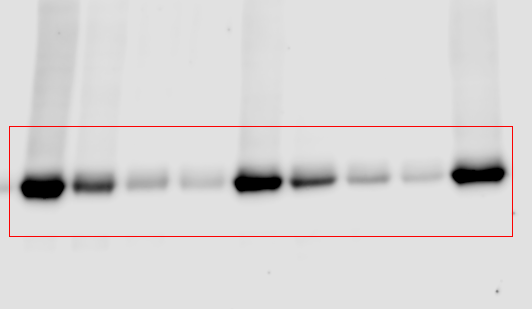

Supplement: Supplementary file 7 — Source data Fig. 5 [file 44318_2025_647_MOESM7_ESM.zip › SD figure 5/5L WB.tif]

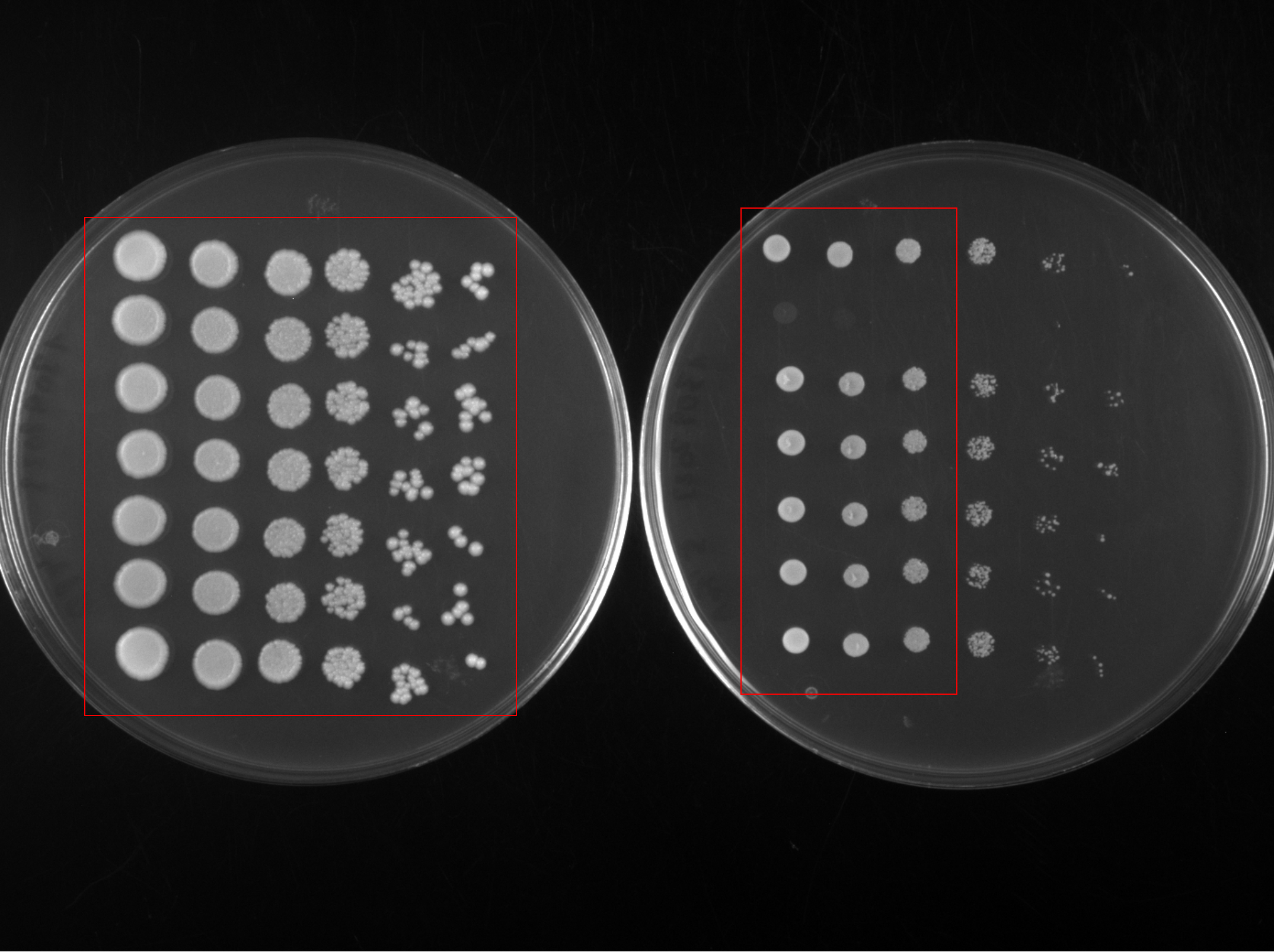

Supplement: Supplementary file 8 — Source data Fig. 6 [file 44318_2025_647_MOESM8_ESM.zip › SD figure 6/6B Drop assay.tif]

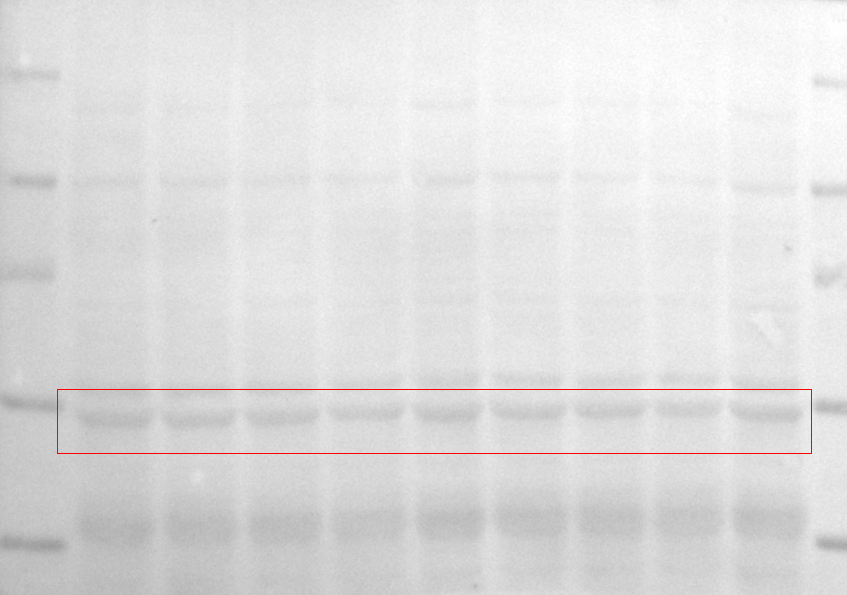

Supplement: Supplementary file 8 — Source data Fig. 6 [file 44318_2025_647_MOESM8_ESM.zip › SD figure 6/6C Ponceau.tif]

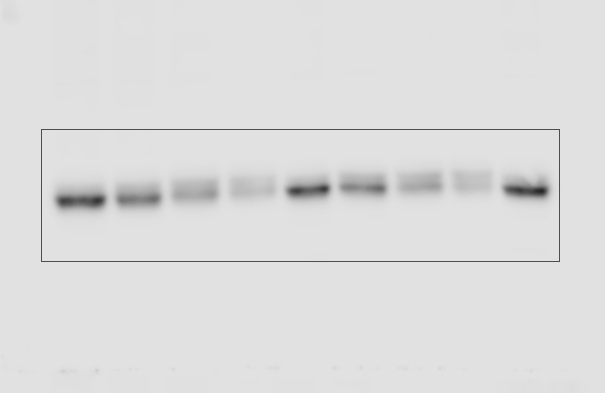

Supplement: Supplementary file 8 — Source data Fig. 6 [file 44318_2025_647_MOESM8_ESM.zip › SD figure 6/6C WB.tif]

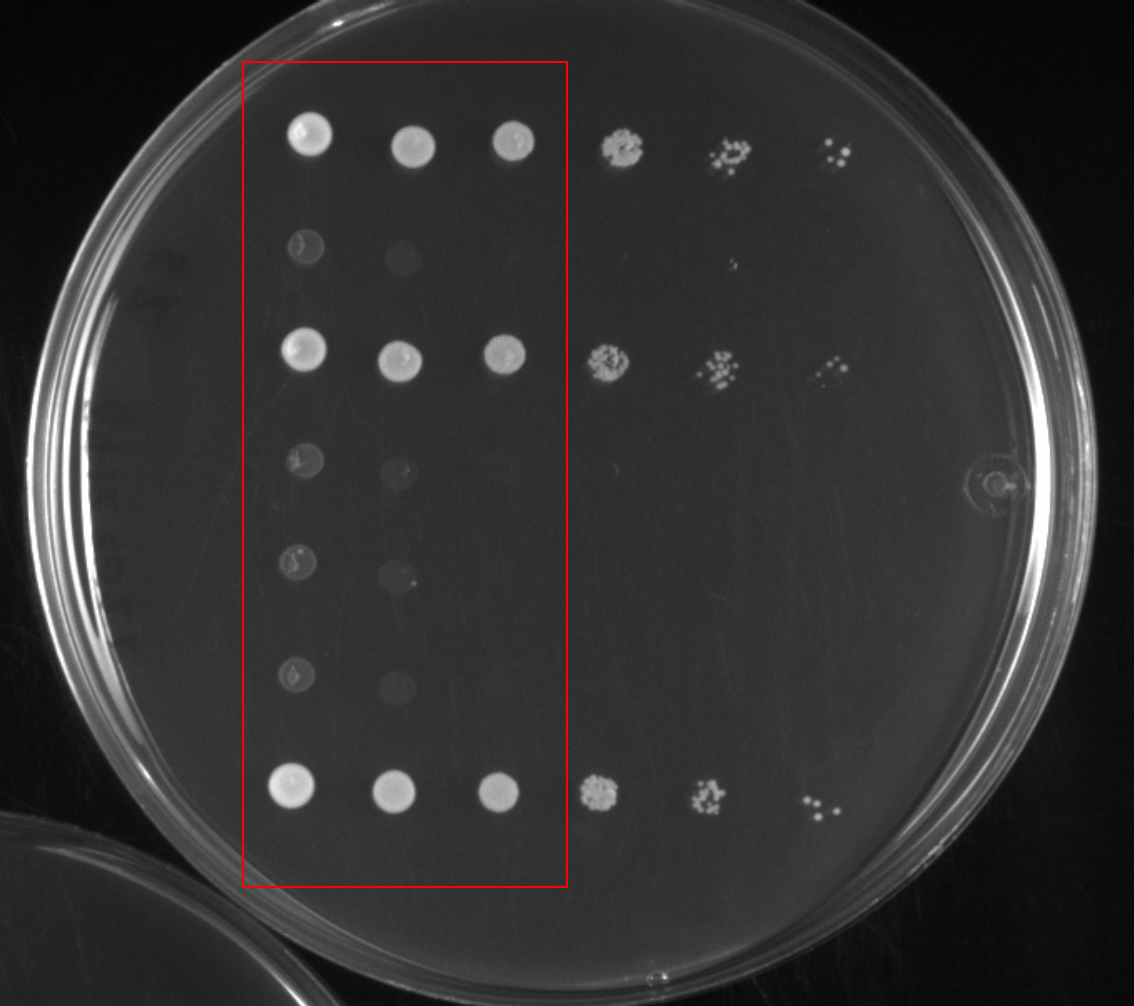

Supplement: Supplementary file 8 — Source data Fig. 6 [file 44318_2025_647_MOESM8_ESM.zip › SD figure 6/6E Drop Assay 5FoA.tif]

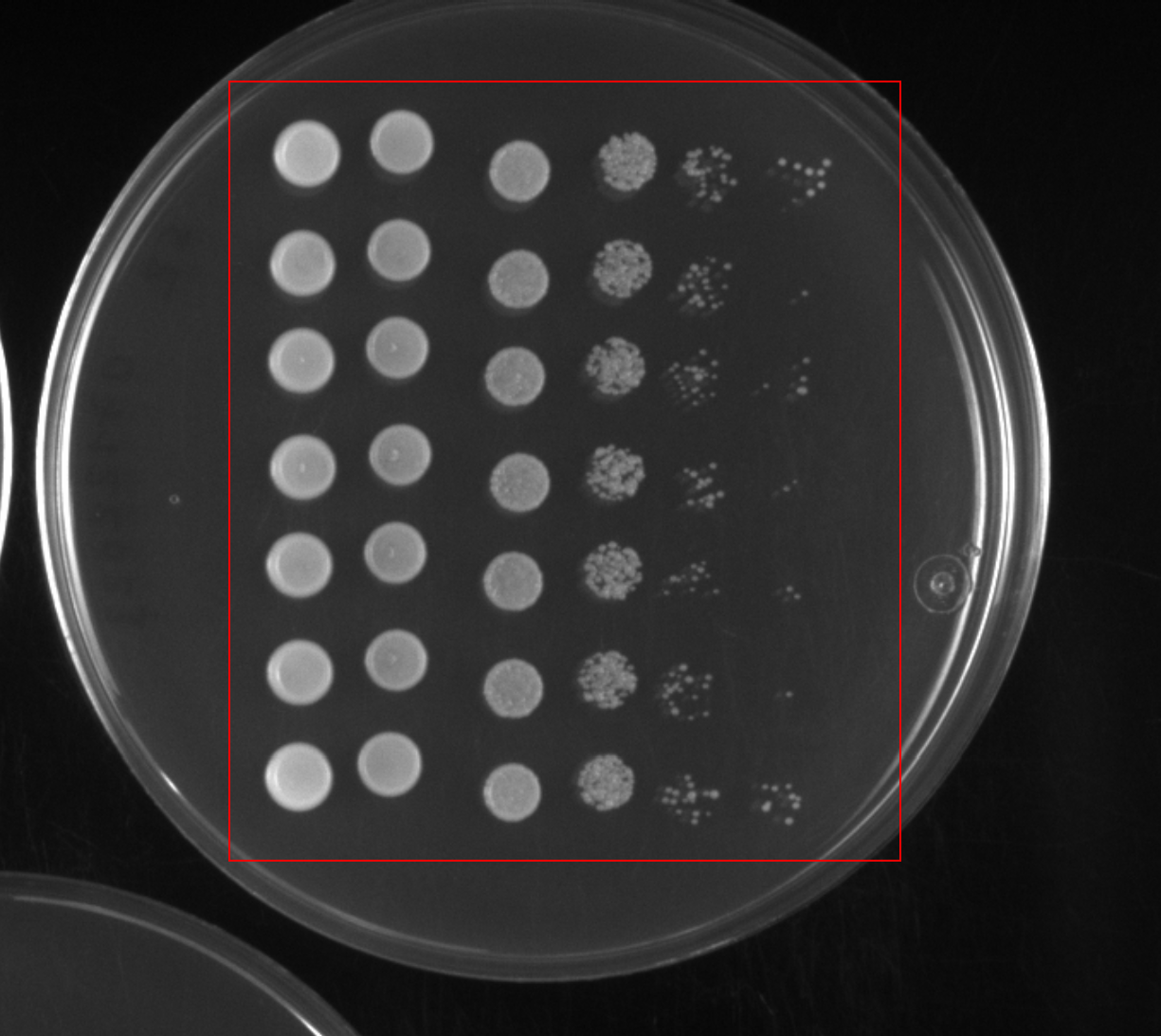

Supplement: Supplementary file 8 — Source data Fig. 6 [file 44318_2025_647_MOESM8_ESM.zip › SD figure 6/6E Drop Assay YPD.tif]

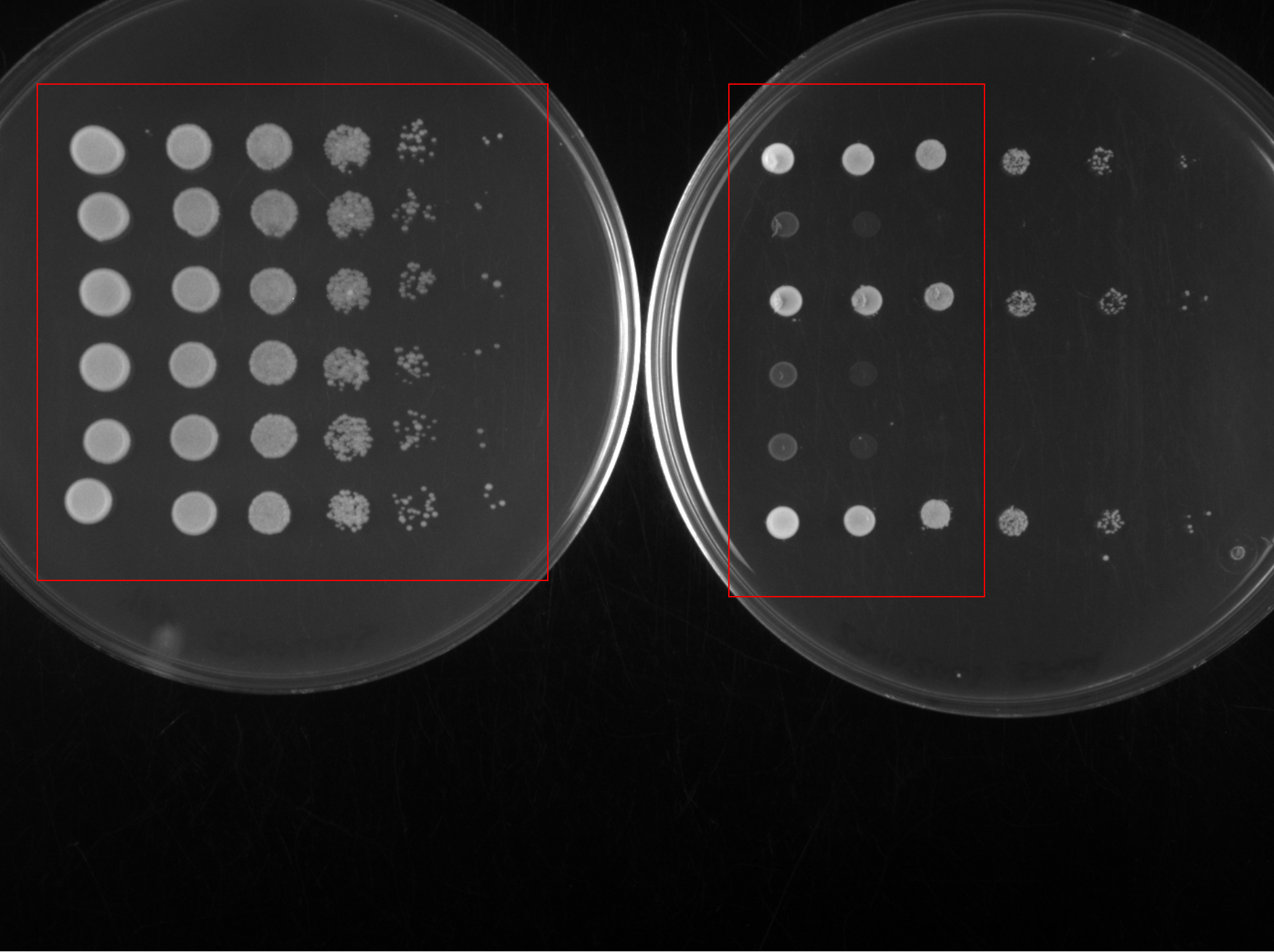

Supplement: Supplementary file 8 — Source data Fig. 6 [file 44318_2025_647_MOESM8_ESM.zip › SD figure 6/6F Drop Assay.tif]

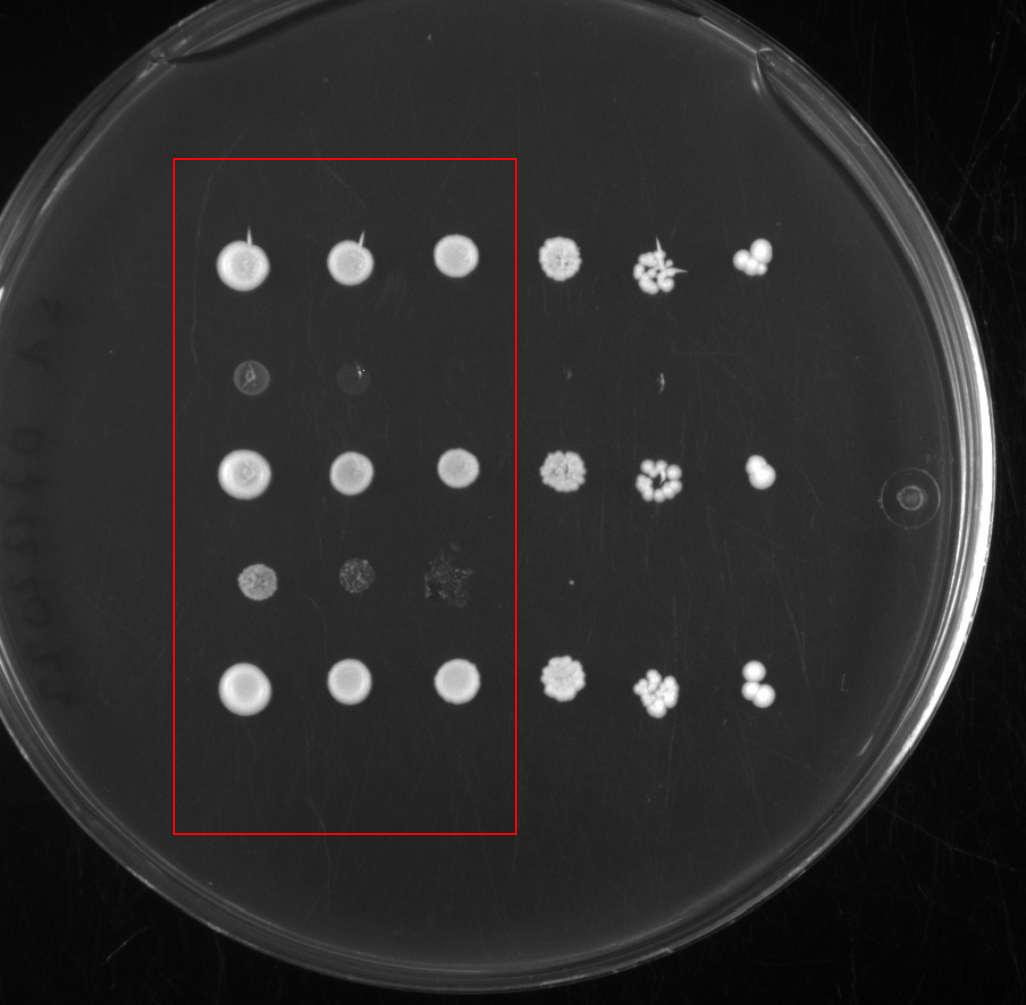

Supplement: Supplementary file 8 — Source data Fig. 6 [file 44318_2025_647_MOESM8_ESM.zip › SD figure 6/6G Drop Assay 5FoA.tif]

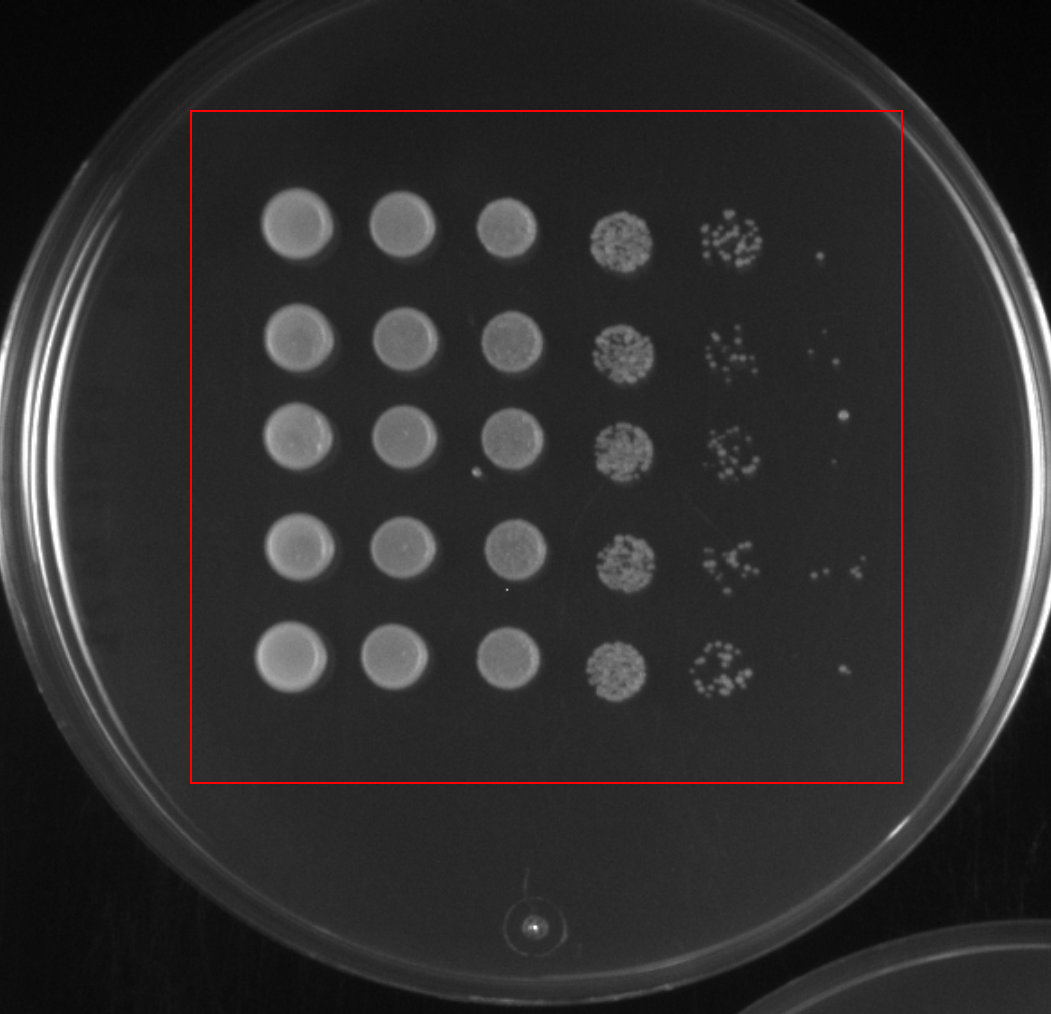

Supplement: Supplementary file 8 — Source data Fig. 6 [file 44318_2025_647_MOESM8_ESM.zip › SD figure 6/6G Drop Assay YPD.tif]

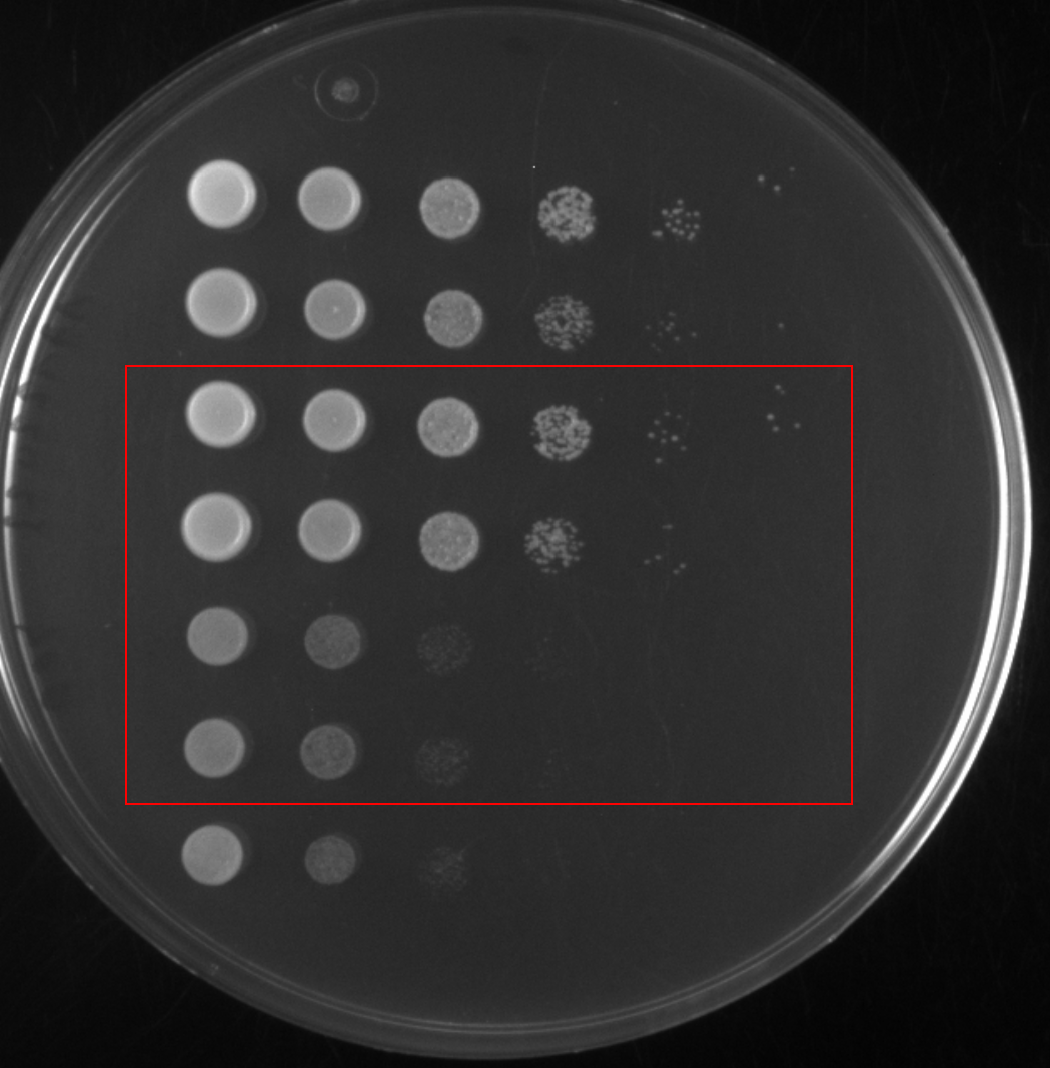

Supplement: Supplementary file 8 — Source data Fig. 6 [file 44318_2025_647_MOESM8_ESM.zip › SD figure 6/6H Drop Assay.tif]

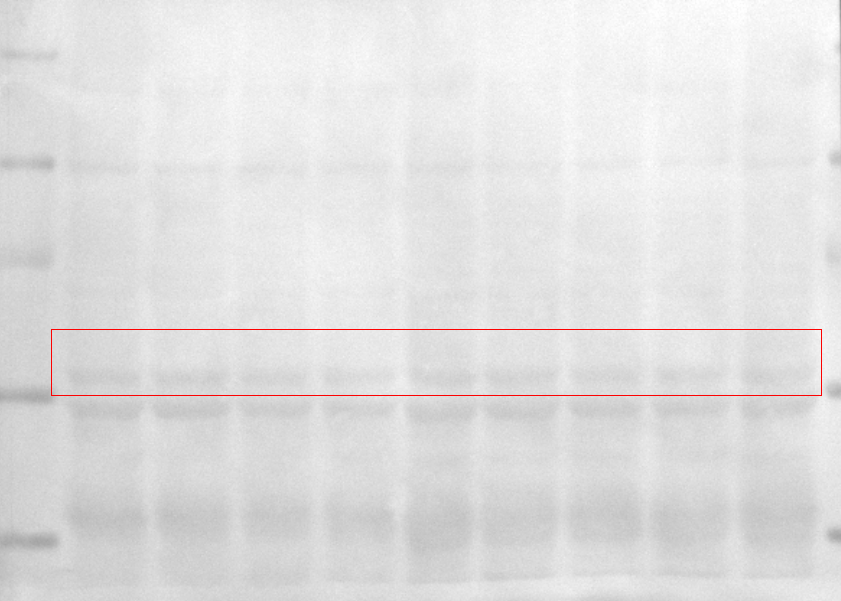

Supplement: Supplementary file 8 — Source data Fig. 6 [file 44318_2025_647_MOESM8_ESM.zip › SD figure 6/6I Ponceau.tif]

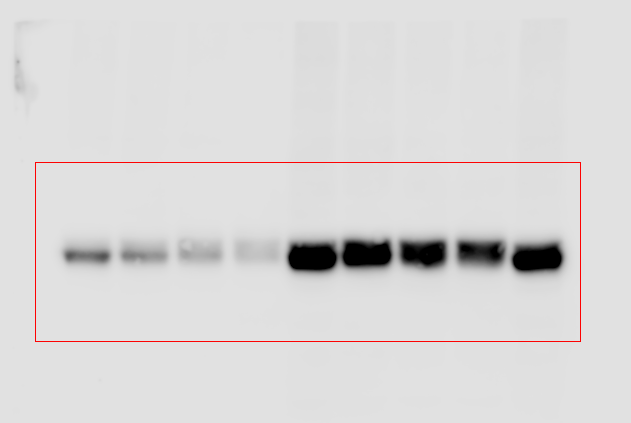

Supplement: Supplementary file 8 — Source data Fig. 6 [file 44318_2025_647_MOESM8_ESM.zip › SD figure 6/6I WB.tif]

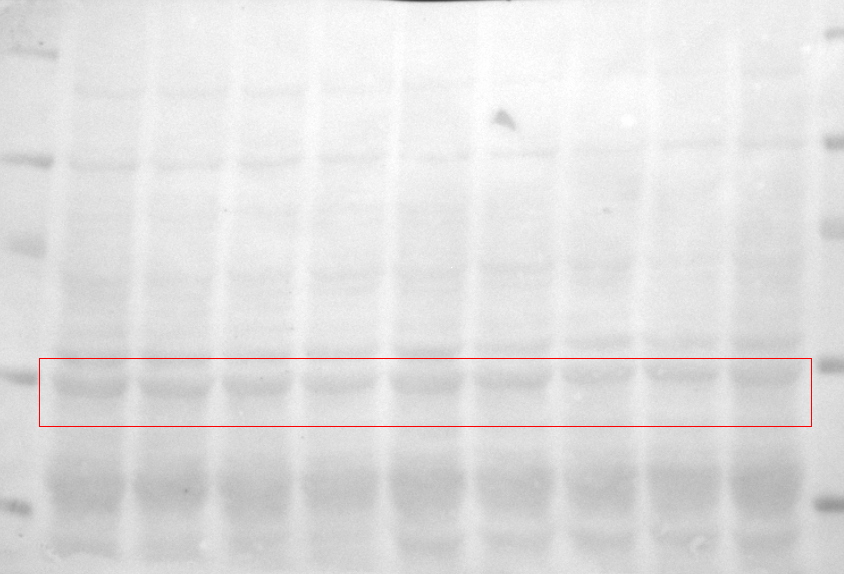

Supplement: Supplementary file 8 — Source data Fig. 6 [file 44318_2025_647_MOESM8_ESM.zip › SD figure 6/6K Ponceau.tif]

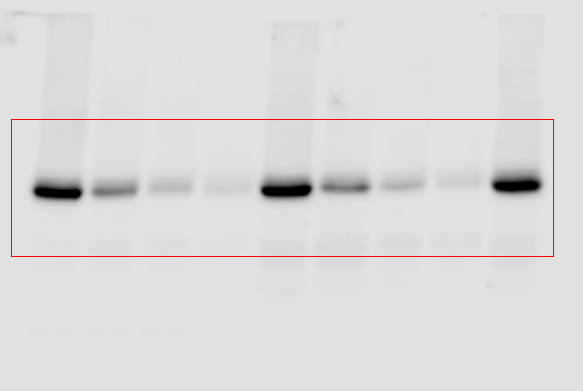

Supplement: Supplementary file 8 — Source data Fig. 6 [file 44318_2025_647_MOESM8_ESM.zip › SD figure 6/6K WB.tif]
