# Supplementary material for: O-mannosylation of misfolded ER proteins promotes ERAD
Source: EMBO J. 2025 Dec 5;45(2):564–91. doi: 10.1038/s44318-025-00647-2 (PMC12811338; doi:10.1038/s44318-025-00647-2)
Supplement: Supplementary file 9 — Expanded View Figures [file 44318_2025_647_MOESM9_ESM.pdf]

Expanded View Figures

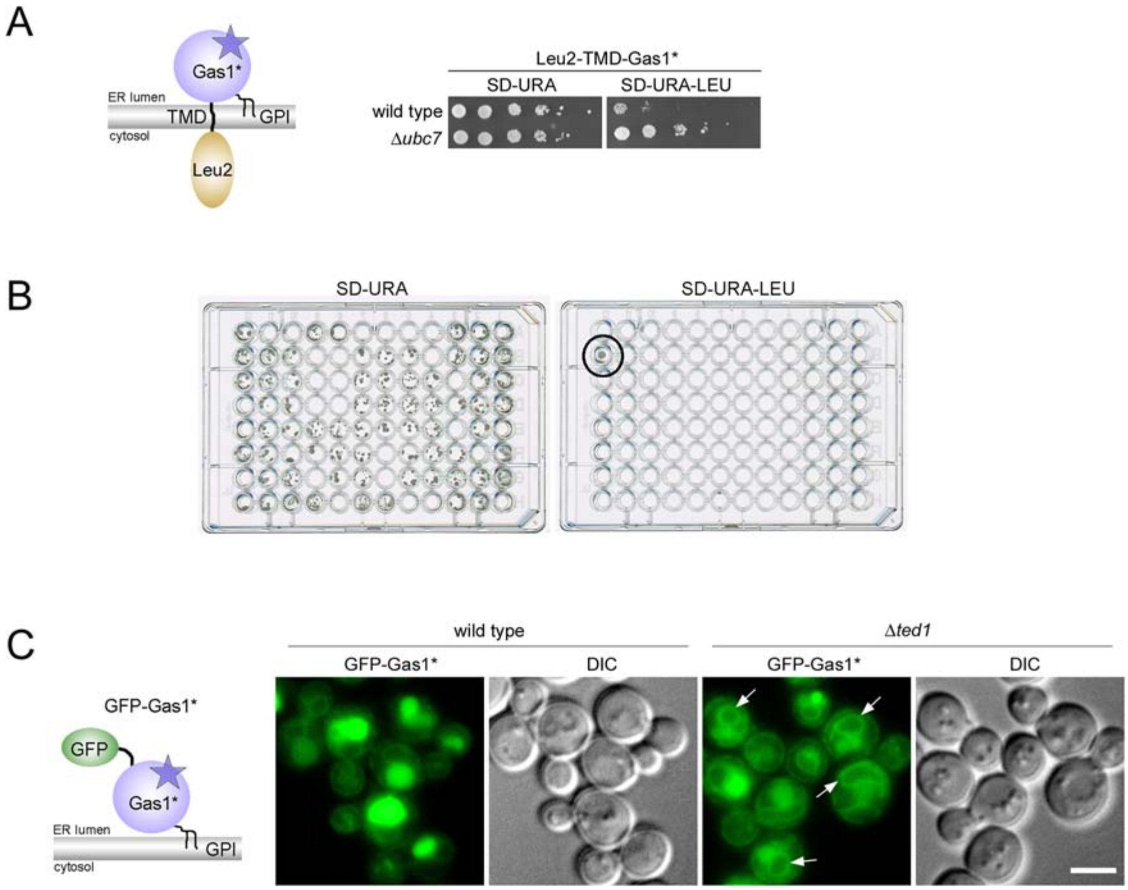

Figure EV1. Setup of complementary genome-wide screens.

(**A**) Design and functionality test of the first screen. Wild-type cells and cells with *UBC7* deleted (*Δubc7*) were used to express the plasmid-borne reporter construct Leu2-TMD-Gas1* (graphical depiction). The plasmid contained *URA3* as a marker and expressed the reporter from the *GAL4* promoter. Cells were grown in medium lacking uracil and spotted in serial dilutions in equal concentration on plates containing synthetic medium lacking uracil (SD-URA) or lacking both uracil and leucine (SD-URA-LEU). Plates were incubated for 3 days (SD-URA) or 5 days (SD-URA-LEU) at 30 °C prior to imaging. The growth of *Δubc7* mutant cells in the absence of leucine (SD-URA-LEU) indicated an impairment in reporter degradation, as expected. (**B**) Screen readout. Representative example for pairs of 96-well plates used for the identification of hits. Transformants of the yeast deletion collection (*Δxxx*) and the collection of decreased abundance by mRNA perturbation (*DAmP*) alleles were first grown in 96-well plates containing liquid synthetic medium lacking uracil (SD-URA). Note that the organization of the libraries is such that not all wells contain cells. After growth in SD-URA, cells were replica-plated into 96-well plates containing medium lacking uracil (SD-URA) and lacking both uracil and leucine (SD-URA-LEU), a pair of plates is shown as example. Growth in both media was considered a hit (circled well). (**C**) Design of the second screen. Wild-type cells and cells with *TED1* deleted (*Δted1*) were used to express genomic N-terminally GFP-tagged Gas1* (graphical depiction) for live-cell high-throughput fluorescence microscopy and differential interference contrast (DIC) microscopy. Ted1 is involved in GPI anchor remodeling. Its deletion was known to reduce the ER export and vacuolar targeting of Gas1* and to increase its routing to ERAD (Sikorska et al, 2016). In agreement, while most GFP was visible inside vacuoles in wild-type cells, indicating efficient targeting of GFP-Gas1* to vacuoles, a significant fraction of the protein accumulated in the perinuclear ER in *Δted1* cells (arrows). Differential interference contrast (DIC) microscopy was used to identify vacuoles. Scale bar: 4 µm.

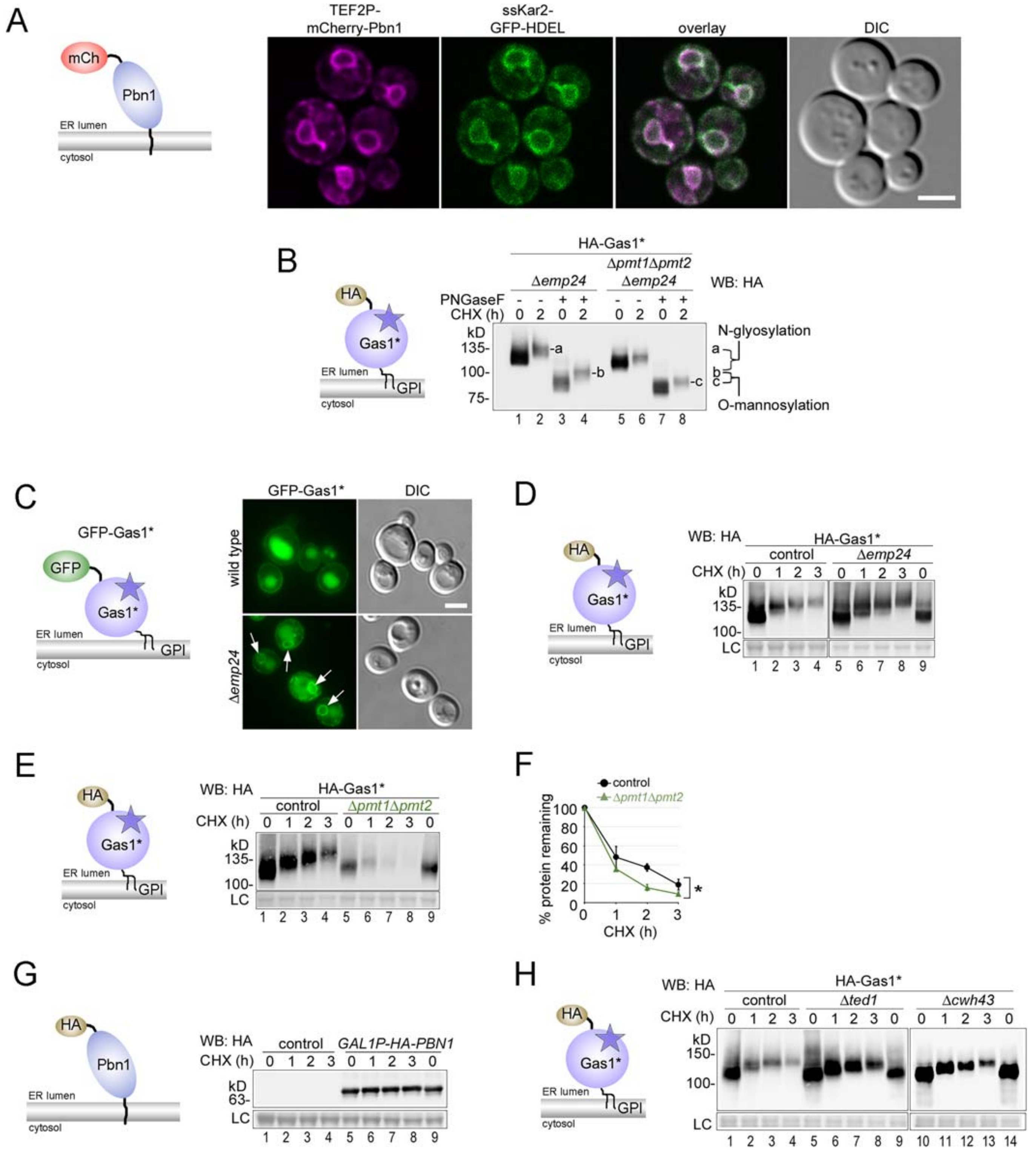
A
mCh
Pbn1
ER lumen
cytosol
TEF2P-
mCherry-Pbn1
ssKar2-
GFP-HDEL
overlay
DIC
B
HA
Gas1*
ER lumen
GPI
cytosol
HA-Gas1*
Δemp24
Δpmt1Δpmt2
Δemp24
WB: HA
PNGaseF - - + + - - + +
CHX (h) 0 2 0 2 0 2 0 2
kD
135-
100-
75-
-a
-b
-c
N-glyosylation
a
b
c
O-mannosylation
1 2 3 4 5 6 7 8
C
GFP-Gas1*
GFP
Gas1*
ER lumen
GPI
cytosol
GFP-Gas1*
DIC
wild type
Δemp24
D
HA
Gas1*
ER lumen
GPI
cytosol
WB: HA
HA-Gas1*
control
Δemp24
CHX (h) 0 1 2 3 0 1 2 3 0
kD
135-
100-
LC
1 2 3 4 5 6 7 8 9
E
HA
Gas1*
ER lumen
GPI
cytosol
WB: HA
HA-Gas1*
control
Δpmt1Δpmt2
CHX (h) 0 1 2 3 0 1 2 3 0
kD
135-
100-
LC
1 2 3 4 5 6 7 8 9
F
control
Δpmt1Δpmt2
% protein remaining
100
80
60
40
20
0
0 1 2 3
CHX (h)
*
G
HA
Pbn1
ER lumen
cytosol
WB: HA
control
GAL1P-HA-PBN1
CHX (h) 0 1 2 3 0 1 2 3 0
kD
63-
LC
1 2 3 4 5 6 7 8 9
H
HA
Gas1*
ER lumen
GPI
cytosol
WB: HA
HA-Gas1*
control
Δted1
Δcwh43
CHX (h) 0 1 2 3 0 1 2 3 0 0 1 2 3 0
kD
150-
100-
LC
1 2 3 4 5 6 7 8 9 10 11 12 13 14

© The Author(s)

Figure EV2. Cellular localization of Pbn1 and Gas1*.

Changes in the O-mannosylation of Gas1* in different mutants. (**A**) Pbn1 localizes to the ER. Yeast cells co-expressing genomic N-terminally mCherry-tagged Pbn1 from the strong constitutive *TEF2* promoter (graphical depiction) and the ER marker ssKar2-GFP-HDEL were analyzed by live-cell fluorescence and DIC microscopy. The degree of colocalization indicated that Pbn1 does not "leak" from the ER, even at a high expression level. Scale bar: 3 µm. (**B**) Gas1* is both N-glycosylated and O-mannosylated. Cells with the indicated deletions were used to express plasmid-borne HA-tagged Gas1* (graphical depiction) for cycloheximide (CHX) shut-off experiments. To prevent rapid ER export and vacuolar degradation of Gas1* caused by deletion of *PMT1* and *PMT2* (*Δpmt1Δpmt2*), the experiment was performed with an additional mutation, in the *Δemp24* background to retain the substrate in the ER, as shown in the fluorescence image ((Fig. EV2C and Goder and Melero, 2011). Cells were lysed at the indicated time points and, where marked (+), lysates were treated with Peptide-N-Glycosidase F (PNGaseF) for the removal of all N-linked glycans, prior to SDS-PAGE and western blot (WB) analysis with anti-HA antibodies. The visible shift in MW after treatment with PNGaseF was reflective of extensive Gas1* N-glycosylation (compare lanes 2 and 4). The additional shift in MW visible in *Δpmt1Δpmt2Δemp24* cells compared to *Δemp24* cells was indicative of protein O-mannosylation (compare lanes 4 and 8). (**C**) Genetic blocking of Gas1* ER export. Wild-type cells and *Δemp24* cells were used to express genomic N-terminally GFP-tagged Gas1* (graphical depiction) for live-cell fluorescence microscopy and DIC microscopy. The fraction of GFP-Gas1* that is targeted to the vacuole is visible due to the accumulation of vacuolar GFP. Deletion of *EMP24* is known to block ER export of Gas1* and to reduce the protein population that is targeted to the vacuole (Sikorska et al, 2016). Arrows indicate accumulated GFP-Gas1* in the ER in *Δemp24* cells. Scale bar: 3 µm. (**D**) Gas1* O-mannosylation occurs inside the ER. Control cells and *Δemp24* cells were used to express plasmid-borne HA-tagged Gas1* for CHX shut-off experiments. Cells were lysed at the indicated time points after the addition of CHX, and the remaining HA-Gas1* was measured by SDS-PAGE and WB with anti-HA antibodies. Membrane staining with Ponceau served as a loading control (LC). The comparable increase in molecular weight (MW) of Gas1* prior to degradation in both control cells and *Δemp24* cells, despite genetically blocked ER exit in the latter, is indicative of extensive protein O-mannosylation occurring within the ER. Stabilization of Gas1* in *Δemp24* cells was reported previously (Sikorska et al, 2016). (**E**) Gas1* degradation is accelerated in the absence of the Pmt1/2-complex. Control cells and *Δpmt1Δpmt2* cells were used to express plasmid-borne HA-tagged Gas1* for CHX shut-off experiments. Cells with *PMT1* and *PMT2* deleted (*Δpmt1Δpmt2*) showed two effects. First, the increase in MW was drastically reduced (compare lanes 1–4 with 5–9), in agreement with the protein being O-mannosylated by the Pmt1/2-complex. Second, Gas1* was degraded faster (compare lanes 1–4 with 5–9, and graph). Faster degradation was previously reported to be caused by a loss of ER retention and increased routing to the vacuole under these conditions (Goder and Melero, 2011; Sikorska et al, 2016). (**F**) Quantification of results shown in (**E**). (**G**) Stability of HA-tagged Pbn1 expressed from the *GAL1* promoter. Control cells and cells expressing genomic N-terminally HA-tagged Pbn1 from the *GAL1* promoter (graphical depiction) were used for CHX shut-off experiments. Cells were grown in medium containing galactose as a carbon source. Overexpressed Pbn1 remained stable throughout the experimental period. This strain background was used for all experiments using the overexpression of HA-Pbn1. The loading control is identical to that shown in Fig. 2E, as different sections of the same membrane were used for both figures. (**H**) No non-O-mannosylated Gas1* was detectable in deletion mutants that impact GPI metabolism. Control cells and cells with the indicated deletions were used to express plasmid-borne HA-tagged Gas1* for CHX shut-off experiment and processed like in (**D**). Stabilization of Gas1* in these mutants is due to blockage of ER export and was reported previously (Sikorska et al, 2016). Data information: Number of experiments (*n*) signifies biological replicates. Error bars in graphs represent the standard deviation from the mean. Statistical significance for experiments involving degradation rates was calculated using two-way ANOVA (alpha = 0.05) with Šidák's correction for multiple comparisons, obtaining the following *p* values. (**F**): ($n = 2$, $p = 0.0103$). Difference in degradation rate: *$p < 0.05$.

© The Author(s)

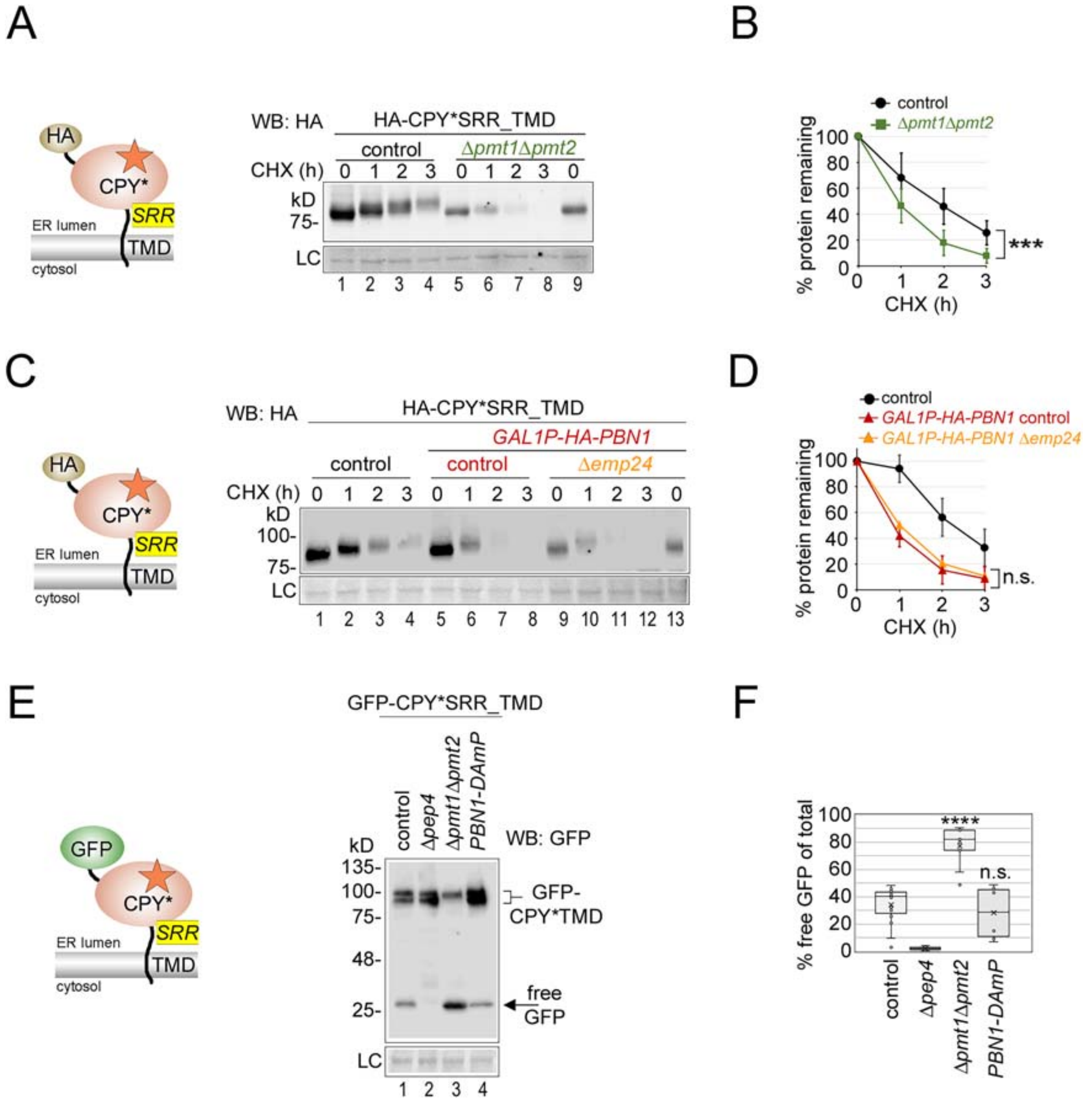
A
HA
CPY*
SRR
ER lumen
TMD
cytosol
WB: HA
HA-CPY*SRR_TMD
control
Δpmt1Δpmt2
CHX (h) 0 1 2 3 0 1 2 3 0
kD
75-
LC
1 2 3 4 5 6 7 8 9
B
control
Δpmt1Δpmt2
% protein remaining
100
80
60
40
20
0
0 1 2 3
CHX (h)
***
C
HA
CPY*
SRR
ER lumen
TMD
cytosol
WB: HA
HA-CPY*SRR_TMD
GAL1P-HA-PBN1
control
control
Δemp24
CHX (h) 0 1 2 3 0 1 2 3 0 1 2 3 0
kD
100-
75-
LC
1 2 3 4 5 6 7 8 9 10 11 12 13
D
control
GAL1P-HA-PBN1 control
GAL1P-HA-PBN1 Δemp24
% protein remaining
100
80
60
40
20
0
0 1 2 3
CHX (h)
n.s.
E
GFP
CPY*
SRR
ER lumen
TMD
cytosol
GFP-CPY*SRR_TMD
control
Δpep4
Δpmt1Δpmt2
PBN1-DAmP
WB: GFP
kD
135-
100-
75-
48-
25-
GFP-
CPY*TMD
free
GFP
LC
1 2 3 4
F
% free GFP of total
100
80
60
40
20
0
****
n.s.
control
Δpep4
Δpmt1Δpmt2
PBN1-DAmP

Figure EV3. Characterization of CPY*SRR_TMD.

(**A**) CPY*SRR_TMD is O-mannosylated by the Pmt1/2-complex. Control cells and cells with *PMT1* and *PMT2* deleted (*ΔpmtΔpmt2*) were used to express plasmid-borne HA-tagged CPY*SRR_TMD (graphically depicted) for CHX shut-off experiments. Cells were lysed at the indicated time points after the addition of CHX, and the remaining protein was measured by SDS-PAGE and WB with anti-HA antibodies. As seen with Gas1*, the increase in MW of CPY*SRR_TMD was reduced in *ΔpmtΔpmt2* cells, indicating that the protein was O-mannosylated by the Pmt1/2-complex (compare lanes 1–4 with 5–9). (**B**) Quantification of results shown in (**A**). (**C**, **D**) CPY*SRR_TMD degradation kinetics was unaffected in *Δemp24* cells upon Pbn1 overexpression. Control cells and cells containing *GAL1P-HA-PBN1* and lacking *EMP24 (Δemp24)* where indicated were used to express plasmid-borne HA-CPY*SRR_TMD (depicted). Cells were grown overnight in medium containing raffinose and then diluted into medium containing galactose for the expression of HA-Pbn1 three hours prior to the application of CHX. Cells were processed as described in (**A**). (**E**) Increased routing of CPY*SRR_TMD to the vacuole in *Δpmt1Δpmt2* cells but not in *PBN1-DAmP* cells. To test the amount of CPY*SRR_TMD routed to the vacuole in different backgrounds, we utilized the well-known GFP-cleavage assay (Klionsky et al, 2021). A GFP-tagged version of CPY*SRR_TMD was generated (graphical depiction), leading to the appearance of (free) GFP in WB analysis after vacuolar degradation of the protein, due to the proteolytic stability of the GFP tag (lane 1, "free GFP"). Free GFP was generated inside the vacuole because it was not generated in the absence of the vacuolar master protease Pep4 (lane 2). The relative amount of free GFP was increased in *Δpmt1Δpmt2* cells, indicative of increased routing of CPY*SRR_TMD to the vacuole (lane 3). These findings support previous data that PMTs promote ER retention in addition to protein O-mannosylation (Goder and Melero, 2011; Sikorska et al, 2016). Cells carrying the *PBN1-DAmP* allele did not show increased routing to the vacuole (lane 4). These results suggest that Pbn1 does not possess ER retention activity. (**F**) Quantification of results shown in (**E**). Box plot displaying the percentage of free GFP relative to the total GFP signal in each lysate. Data information: Number of experiments (n) signifies biological replicates. Error bars in graphs represent the standard deviation from the mean. Statistical significance for all experiments involving degradation rates was calculated using two-way ANOVA (alpha = 0.05) with Šidák's correction for multiple comparisons, obtaining the following p values. (**B**): ($n = 9$, $p = 0.0006$), ***$p < 0.001$; (**D**): ($n = 2$, $p = 0.9998$), n.s. = not significant. (**F**) Statistical significance of changes of free GFP in *Δpmt1Δpmt2* and *PBN1-DAmP* cells compared to control cells was determined using an unpaired two-tailed Student's t test. *Δpmt1Δpmt2*: $n = 9$, $p = 2.96 \times 10^{-7}$; *PBN1-DAmP*: $n = 6$, $p = 0.4373$). n.s. = not significant; ****$p < 0.0001$. Numerical values of the box plot shown in (**F**): control: (minimum: 3.1; maximum: 48.3; median: 40.5; Q1: 27.9; Q3: 43.3; lower whisker: 9.9; upper whisker: 48.3); *Δpep4*: (minimum: 0.4; maximum: 4.5; median: 2.1; Q1: 1.3; Q3: 3.2; lower whisker: 0.4; upper whisker: 4.5); *Δpmt1Δpmt2*: (minimum: 49; maximum: 91; median: 82; Q1: 74; Q3: 89; lower whisker: 58; upper whisker: 91); *PBN1-DAmP*: (minimum: 7; maximum: 49; median: 29; Q1: 11; Q3: 45; lower whisker: 7; upper whisker: 49).

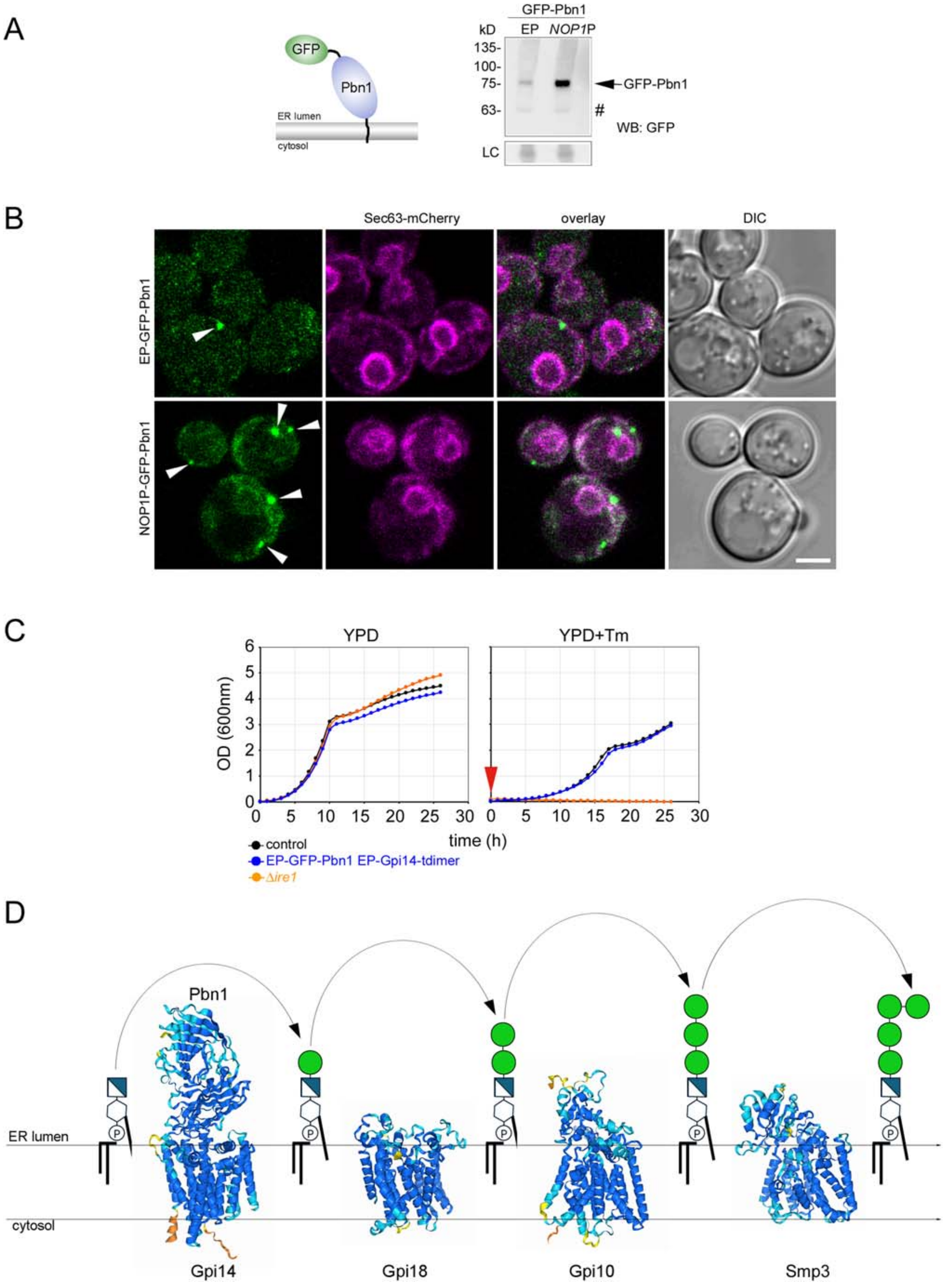
A
GFP
Pbn1
ER lumen
cytosol
GFP-Pbn1
kD
EP
NOP1P
135-
100-
75-
63-
GFP-Pbn1
#
WB: GFP
LC
B
Sec63-mCherry
overlay
DIC
EP-GFP-Pbn1
NOP1P-GFP-Pbn1
C
YPD
YPD+Tm
OD (600nm)
time (h)
control
EP-GFP-Pbn1 EP-Gpi14-tdimer
Δire1
D
Pbn1
ER lumen
cytosol
Gpi14
Gpi18
Gpi10
Smp3

Figure EV4. Properties of Pbn1 and Gpi14.

(**A**) Comparison of cellular levels of GFP-tagged Pbn1 expressed from different promoters. Expression of genomic N-terminally GFP-tagged Pbn1 (graphical depiction) from either the endogenous promoter (EP) or from the moderate *NOP1* promoter (NOP1P). Equal amounts of cells were taken for lysis, and expression levels were compared by SDS-PAGE followed by WB analysis with antibodies against GFP. Hashtag indicates an unspecific band. The results indicated weak expression of GFP-Pbn1 from its endogenous promoter. (**B**) Cellular localization of GFP-Pbn1. Cells co-expressing genomic GFP-Pbn1 from different promoters like in (**A**) and the ER marker Sec63-mCherry were analyzed by live-cell confocal fluorescence microscopy in combination with DIC microscopy. Note that N-terminal tagging of Pbn1 with GFP led to the concentration of the protein in cellular puncta (arrowheads) regardless of protein expression levels. Co-localization with the ER marker Sec63 suggested that Pbn1 puncta localized to the ER. Scale bar: 2 µm. (**C**) Functionality tests of tagged versions of Pbn1 and Gpi14. Wild-type cells (control) and strains expressing genome-integrated fluorescently tagged Pbn1 and Gpi14 were grown in complete synthetic media (SD) in the absence or presence of 1 µg/ml tunicamycin (Tm) at 30 °C. The drug was applied immediately after starting the experiment by resetting cellular density (red arrowhead). Growth was determined by automated measuring of the absorbance of the individual cell cultures at 600 nm over a period of 25 h. Cells with *IRE1* deleted (*Δire1*) are known to be sensitive to the induction of ER stress with tunicamycin and were used as a control. The strains co-expressing GFP-Pbn1 and Gpi14-tdimer showed no growth defect in SD medium in the absence or presence of tunicamycin compared to the control strain. Since both proteins, Pbn1 and Gpi14, are essential genes, these results indicate that the utilized tagging did not compromise the essential functions of either protein. (**D**) Schematic illustrating the four different steps of mannosylation of the GPI precursor and the enzymes involved. The membrane-embedded precursor GlcN-(acyl)PI is mannosylated (green filled circles) in a series of reactions involving the enzymes Pbn1-Gpi14, Gpi18, Gpi10, and Smp3, as depicted. The enzyme structure predictions were obtained with Alphafold3. Additional modifications, such as the addition of ethanolamine phosphate, are not shown. The substrates are not drawn to scale in relation to the enzymes. Note that Pbn1 provides an unusually large ER luminal domain to Gpi14, whereas the other enzymes either lack (Gpi18) or have considerably smaller ER luminal domains (Gpi10 and Smp3), although a similar GPI precursor structure is modified in each case.

© The Author(s)

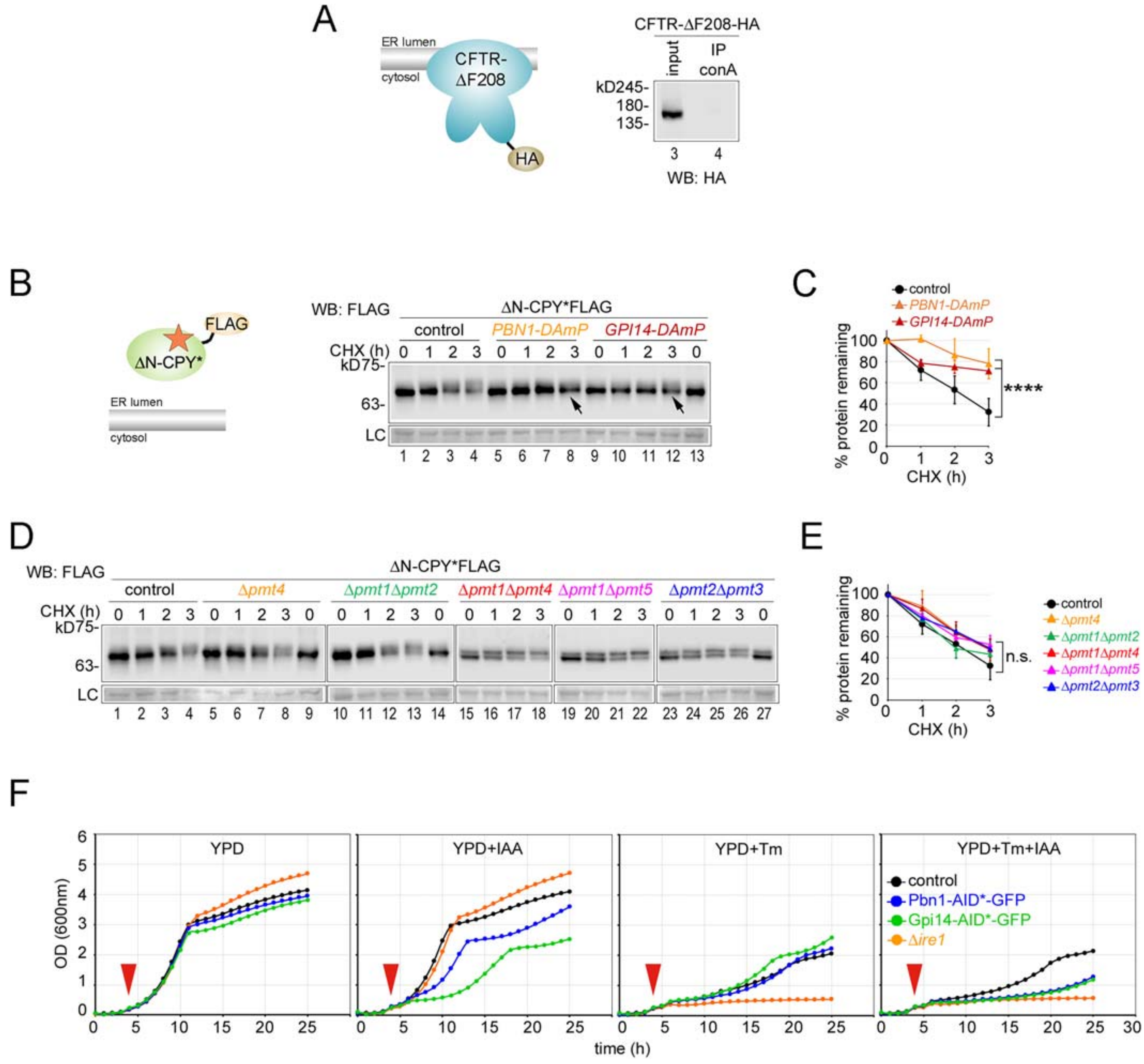
A
ER lumen
cytosol
CFTR-ΔF208
HA
CFTR-ΔF208-HA
input
IP conA
kD245-
180-
135-
3 4
WB: HA
B
FLAG
ΔN-CPY*
ER lumen
cytosol
WB: FLAG
ΔN-CPY*FLAG
control
PBN1-DAmP
GPI14-DAmP
CHX (h) 0 1 2 3 0 1 2 3 0 1 2 3 0
kD75-
63-
LC
1 2 3 4 5 6 7 8 9 10 11 12 13
C
control
PBN1-DAmP
GPI14-DAmP
% protein remaining
CHX (h)
****
D
WB: FLAG
ΔN-CPY*FLAG
control
Δpmt4
Δpmt1Δpmt2
Δpmt1Δpmt4
Δpmt1Δpmt5
Δpmt2Δpmt3
CHX (h)
kD75-
63-
LC
E
% protein remaining
CHX (h)
n.s.
control
Δpmt4
Δpmt1Δpmt2
Δpmt1Δpmt4
Δpmt1Δpmt5
Δpmt2Δpmt3
F
YPD
YPD+IAA
YPD+Tm
YPD+Tm+IAA
OD (600nm)
time (h)
control
Pbn1-AID*-GFP
Gpi14-AID*-GFP
Δire1

 © The Author(s)

Figure EV5. Functional characterization of Pbn1-Gpi14.

(**A**) Control for concanavalin-A-mediated pulldown. Cells expressing plasmid-borne CFTR-ΔF208-HA (graphical depiction) were lysed, and the lysate was incubated with concanavalin-A-coupled beads (IP conA) for 3 h, followed by SDS-PAGE and WB analysis with antibodies against HA. CFTR-ΔF208-HA is not glycosylated in yeast, and its failure to be precipitated with conA served as a specificity control for the assay and validated the results obtained with ΔNg-CPY* (Fig. 5G). (**B**, **C**) Control cells and cells containing the *PBN1-DAmP* or *GPI14-DAmP* alleles were used to express plasmid-borne ΔNg-CPY*FLAG (graphical depiction) for CHX shut-off experiments, followed by SDS-PAGE and WB analysis. The arrows indicate an accumulation of ΔNg-CPY*FLAG with lower MW compared to the control. (**D**, **E**) Experiments using PMT deletion mutants. Yeast expresses six verified PMTs, with the most well-characterized being the conserved Pmt1, Pmt2, and Pmt4 (Lommel and Strahl, 2009). Control cells and cells with the indicated single and double deletions of PMT genes were used to express plasmid-borne ΔNg-CPY*FLAG for CHX shut-off experiments. Neither in the *Δpmt4* mutant, which lacks the homodimeric Pmt4 complex, nor in the *Δpmt1Δpmt2* double mutant, which lacks the heterodimeric Pmt1/2 complex, did we detect alterations in O-mannosylation of ΔNg-CPY* (lanes 1–14). The same results were obtained with those double mutants that simultaneously prevented the formation of the canonical and a cross-combinatorial Pmt1/2 complex (lanes 15–27). These results combined suggest that canonical PMTs do not participate in the O-mannosylation and degradation of ΔNg-CPY*. (**F**) Growth assays in the absence and presence of ER stress after acute depletion of either Pbn1 or Gpi14. The indicated strains were grown in rich medium (YPD) in the absence or presence of 1 μg/ml tunicamycin (Tm) and 500 μM auxin (indole-3-acetic acid (IAA)) at 30 °C. Drugs together with solvents or solvents alone were applied 4 h after starting the experiment by resetting cellular density (indicated by red arrowheads). Growth was determined by automated measurement of absorbance at 600 nm for individual cell cultures over a period of 25 h. The values are the mean of two independent measurements. Data information: Number of experiments (n) signifies biological replicates. Error bars in graphs represent the standard deviation from the mean. Statistical significance for all experiments involving degradation rates was calculated using two-way ANOVA (alpha = 0.05) with Šidák's correction for multiple comparisons, obtaining the following p values. (**C**): (*PBN1-DAmP*: $n = 4$, $p = 0.9 \times 10^{-4}$; *GPI14-DAmP*: $n = 4$, $p = 0.9 \times 10^{-4}$), (**E**): (*Δpmt4*: $n = 2$, $p = 0.6803$; *Δpmt1Δpmt2*: $n = 4$, $p = 0.9260$; *Δpmt1Δpmt4*: $n = 4$, $p = 0.2865$; *Δpmt1Δpmt5*: $n = 2$, $p = 0.8328$; *Δpmt2Δpmt3*: $n = 2$, $p = 0.5133$). n.s. = not significant; ****$p < 0.0001$.

© The Author(s)

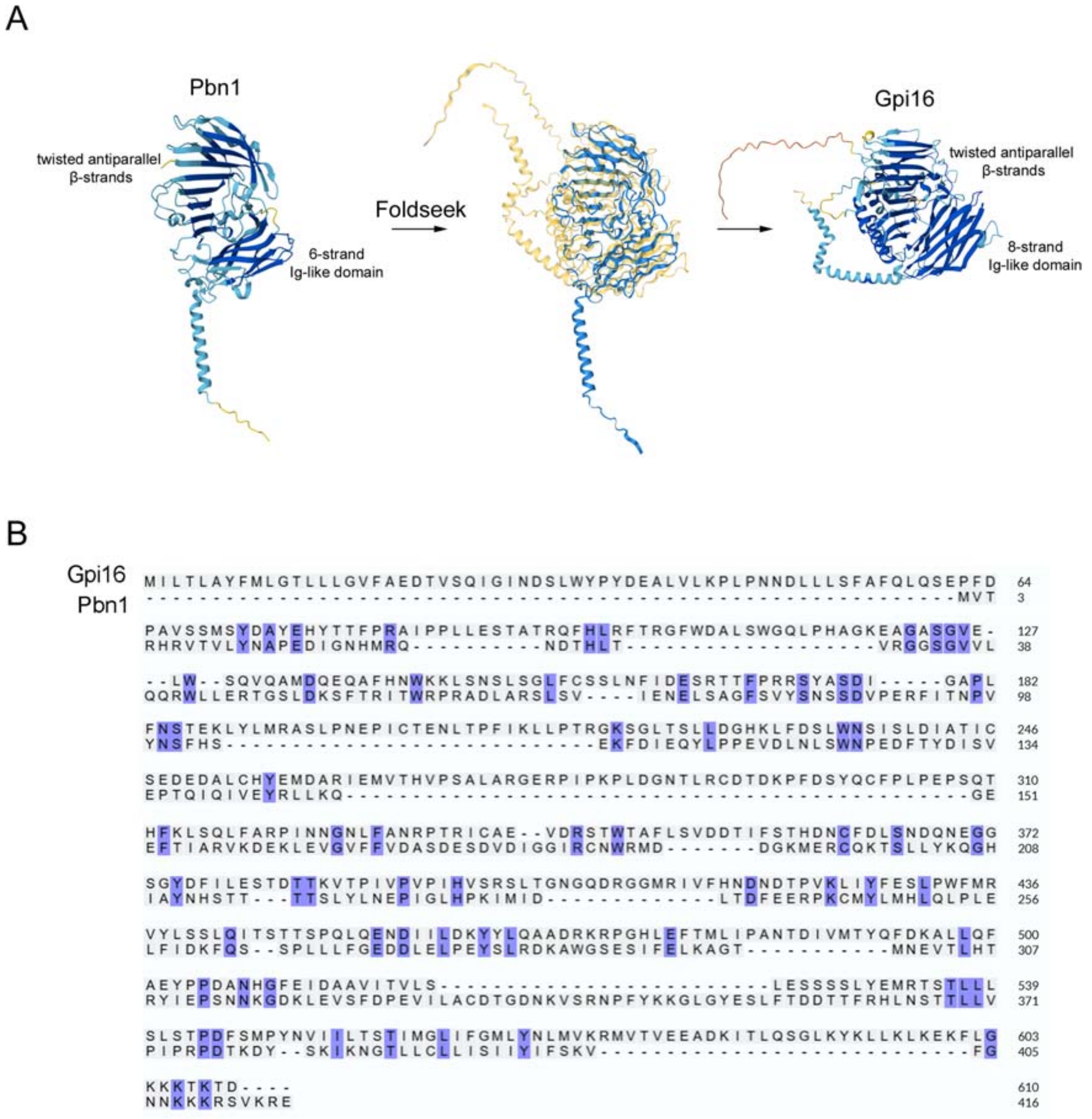

Figure EV6. Search for proteins with similar folds to Pbn1.

(**A**) Identification of Gpi16 as a protein structurally similar to Pbn1. Foldseek (van Kempen et al, 2024) was employed to identify structural similarities across proteins from all kingdoms using the Pbn1 Alphafold3 structure as bait (left). The essential component of the pentameric GPI-transamidase complex, Gpi16, emerged as the sole hit with a high degree of similarity. This is illustrated by the overlay (yellow) in the center. The Alphafold3 structure of Gpi16 is displayed on the right. (**B**) Low sequence identity between Pbn1 and Gpi16. Protein alignment analysis revealed a low overall sequence identity (18%) between Pbn1 and Gpi16. Conserved amino acids are highlighted in purple.

 © The Author(s)
